# Impact and Control of Sugar Size in Glycoconjugate Vaccines

**DOI:** 10.3390/molecules27196432

**Published:** 2022-09-29

**Authors:** Giuseppe Stefanetti, Calman Alexander MacLennan, Francesca Micoli

**Affiliations:** 1 Department of Biomolecular Sciences, University of Urbino Carlo Bo, 61029 Urbino, Italy; 2 Enteric and Diarrheal Diseases, Global Health, Bill & Melinda Gates Foundation, 500 5th Ave. N, Seattle, WA 98109, USA; 3 The Jenner Institute, Nuffield Department of Medicine, University of Oxford, Oxford OX3 7DQ, UK; 4 The Institute of Immunology and Immunotherapy, University of Birmingham, Birmingham B15 2TT, UK; 5 GSK Vaccines Institute for Global Health, 53100 Siena, Italy

**Keywords:** vaccine, glycoconjugates, immunogenicity, sugar length, fragmentation of polysaccharides

## Abstract

Glycoconjugate vaccines have contributed enormously to reducing and controlling encapsulated bacterial infections for over thirty years. Glycoconjugate vaccines are based on a carbohydrate antigen that is covalently linked to a carrier protein; this is necessary to cause T cell responses for optimal immunogenicity, and to protect young children. Many interdependent parameters affect the immunogenicity of glycoconjugate vaccines, including the size of the saccharide antigen. Here, we examine and discuss the impact of glycan chain length on the efficacy of glycoconjugate vaccines and report the methods employed to size polysaccharide antigens, while highlighting the underlying reaction mechanisms. A better understanding of the impact of key parameters on the immunogenicity of glycoconjugates is critical to developing a new generation of highly effective vaccines.

## 1. Introduction

Many bacteria are surrounded by capsular polysaccharides (CPS) or O-specific polysaccharides (O-SP), which are both major virulence factors and important targets for protective antibodies [1,2]. Since the 1930s, the protective role of antibodies induced by pneumococcal CPS began to be investigated, and in 1945, the first vaccine composed of purified CPS from selected pneumococcal serotypes was tested on man [3,4]. Between the 1970s and 1980s, polysaccharide (PS) vaccines against the meningococcal serogroup (Men) ACWY, *Streptococcus pneumoniae*, and *Haemophilus influenzae* type b (Hib), were developed. Although these vaccines have shown variable degrees of success in adults, they are not especially protective for high-risk populations, particularly children below two years and immunocompromised patients [5]. PSs are high molecular weight (MW) biopolymers characterized by repeating antigenic epitopes that are spatially close to each other and they can cross-link multiple surface immunoglobulins expressed on B cells. PSs are T-independent antigens that are able to directly activate PS-specific B cells which can then differentiate into plasma cells and produce antibodies; however, memory B cells are not formed. Re-vaccination, which is frequently required as antibody levels decline rapidly, does not elicit a booster effect. Indeed, immunization with unconjugated PSs can deplete a preexisting memory B-cell pool, leading to hyporesponsiveness to subsequent immunization. Additionally, the antibody response to PSs is little affected by adjuvants [6,7].

These drawbacks of the immune response, induced by pure PSs, can be overcome by glycoconjugate vaccines, consisting of a PS covalently linked to an appropriate carrier protein as source of T-cell epitopes [8,9]. Conjugation confers to the saccharide the capacity to induce a long-lasting and boostable IgG antibody response, even in children under two years of age. Glycoconjugate vaccines have been shown to be more effective in adult populations than PS vaccines. The concept of glycoconjugate vaccines has its origin in 1929, when Avery and Goebel demonstrated that derivatives of non-immunogenic glucose and galactose, if conjugated to proteins, were able to induce specific antibodies in rabbits [10,11]. The first PS-protein conjugate vaccines were developed against Hib and licensed between 1987 and 1990 [12]. Subsequently, glycoconjugate vaccines have been licensed against *Neisseria meningitidis*, *S. pneumoniae*, *Salmonella* Typhi, and many others are currently under development [13,14].

Traditionally, conjugate formation requires the covalent binding of the PS to the carrier protein [15]. In the case of long PSs, the sugar is often randomly—i.e., non-specifically—activated along the chain and linked to the protein. Moreover, when shorter saccharides are used, protein coupling generally occurs through the activation of the saccharide terminal group [16]. Linkers are often used to reduce steric hindrance, thus facilitating the linkage between the two moieties. The random approach results in cross-linked glycoproteins with variable saccharide loading and antigen positioning. The selective approach provides more homogeneous and well-defined molecules, which also benefit the characterization process.

Several parameters can affect the immunogenicity of glycoconjugate vaccines [17], including the size of the saccharide and its structural modifications, the carrier protein used [18], and the saccharide to protein ratio [16]. In addition, the conjugation chemistry, including the presence of the linker and the attachment point on the carrier protein [19], can play an important role. 

In this review, we focus our attention on saccharide chain length by reporting preclinical and clinical evidence concerning the impact of this variable on the immunogenicity of glycoconjugate vaccines. In addition, the methods used to size PS antigens and the underlying reaction mechanisms are also reported.

## 2. Role of Sugar Length on the Immunogenicity of Glycoconjugate Vaccines

Traditionally, for the synthesis of glycoconjugate vaccines, PSs are first extracted from a bacterial culture and isolated through a series of purification steps, which differ according to the nature of the carbohydrate. Depending on the genetic repertoire, bacterial strains can produce PSs of different chain lengths. Furthermore, the fermentation and purification conditions employed can have a profound impact on the length of the sugar chains. According to their nature, PSs are polymers of identical repeating units (RU) (from ten to a few thousand units in length). Although there is a specific “stop” codon in protein synthesis, to ensure that all protein molecules produced are identical, there is no such signal in bacterial PS production; therefore, PS molecules are characterized by a broad range of sizes (i.e., they are polydisperse). The molecular size distribution of each batch of PSs must be determined and monitored, since any variations could indicate that there has been a lack of production consistency or a loss of PS structural integrity during the purification step.

The use of shorter oligosaccharides (OS) has often been preferred to improve conjugation yields and consistency, and facilitate conjugate purification, particularly from unreacted saccharide. In addition, dealing with smaller sized sugars may help characterization throughout the entire process (including sugar activation(s) and conjugation), and it could be beneficial for the production of conjugates with better defined structures [20]. For this reason, PSs have often been chemically or mechanically fragmented, and size-fractionated, to generate homogeneous populations of lower MWs before conjugation to the carrier protein. Many studies have investigated the role that PS length can have on the immune response elicited by corresponding glycoconjugates, and these are reported below.

### 2.1. Dextrans

Seppala and Makela found that low MW Dextrans (Dx) (1–4 kDa), conjugated to chicken serum albumin (CSA) via their reducing end, induced higher anti-Dx antibody responses in mice than 40 kDa Dx [21]. Fernandez and Sverremark reported that 1 kDa Dx, coupled to CSA through their reducing end, gave a greater secondary immune response than 40 kDa Dx conjugated to keyhole limpet hemocyanin (KLH) by multi-point attachment [22].

### 2.2. Group B Streptococcus 

**Figure 1:**
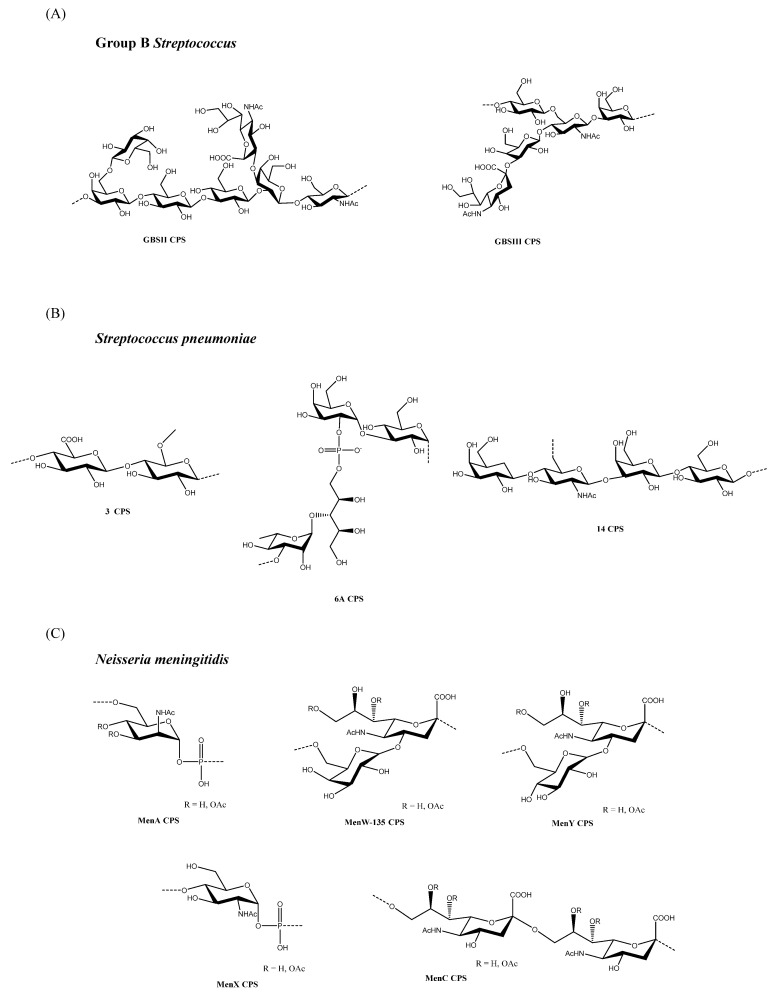

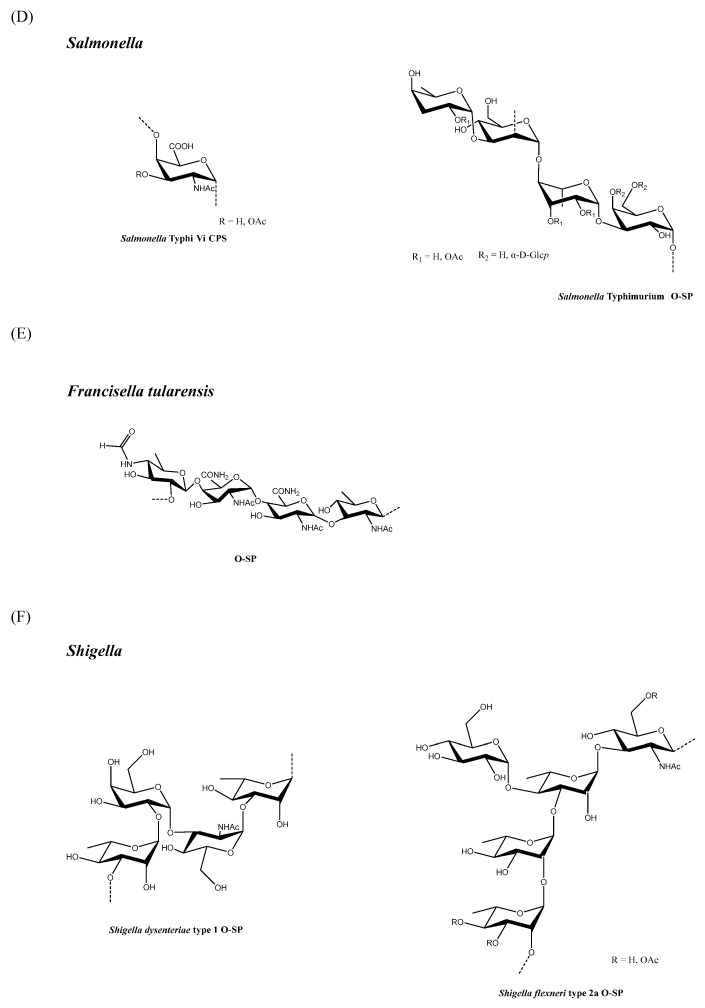

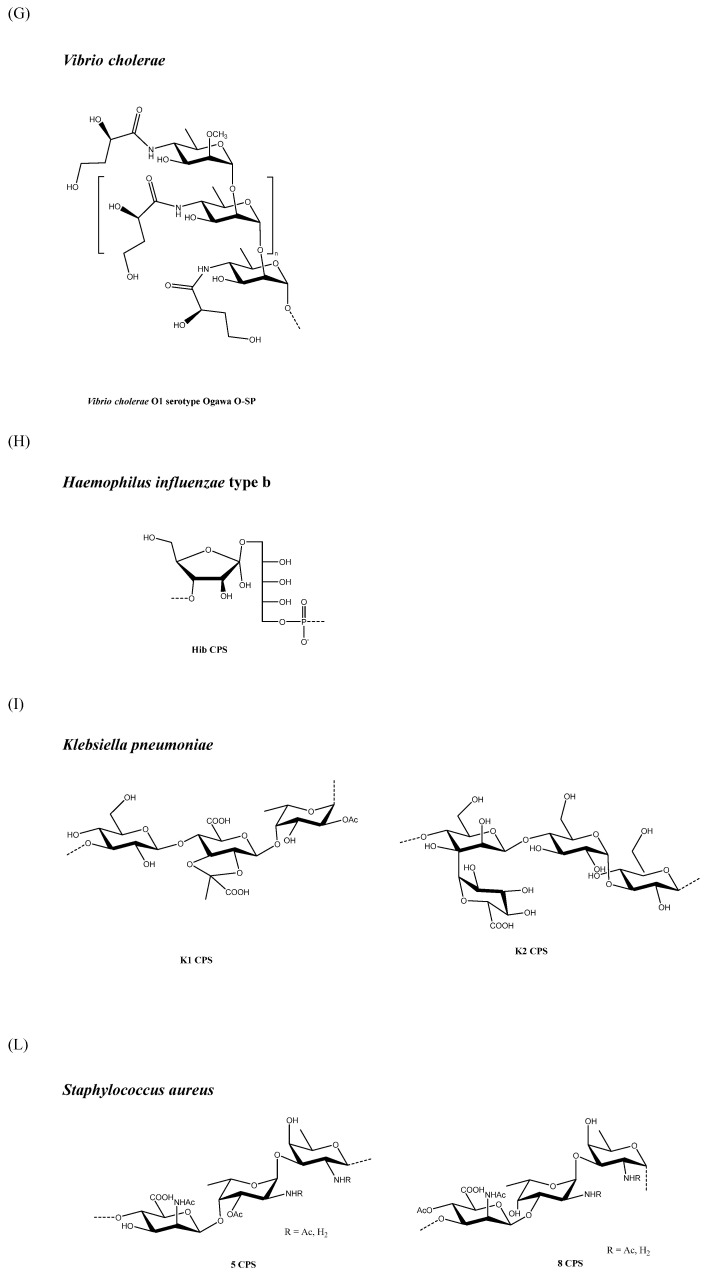

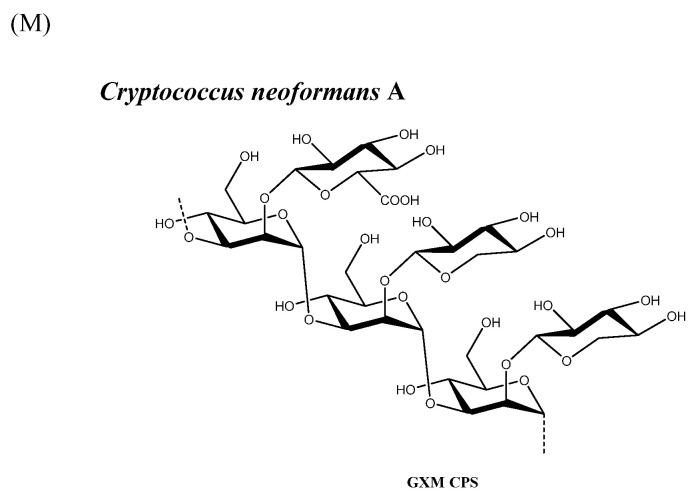
Chemical structure of the PS RU discussed in this review. (**A**) Group B *Streptococcus,* (**B**) *Streptococcus pneumoniae,* (**C**) *Neisseria meningitidis*, (**D**) *Salmonella*, (**E**) *Francisella tularensis*, (**F**) *Shigella*, (**G**) *Vibrio cholerae*, (**H**) *Haemophilus influenzae* type b, (**I**) *Klebsiella pneumoniae*, (**L**) *Staphylococcus aureus*, (**M**) *Cryptococcus neoformans* A.

Paoletti et al. coupled the reducing end of type III Group B *Streptococcus* (GBS) OS of 7, 14.5, and 27 kDa to tetanus toxoid (TT) using an aminoalkyl glycoside spacer [23,24]. All the OS-TT conjugates, having a comparable saccharide to protein molar ratio, induced similar antigen-specific IgG titers in rabbits; however, vaccination with the intermediate-sized (14.5 kDa OS) conjugate resulted in superior in vitro killing of GBS by human blood leukocytes and improved protection against the GBS challenge. Wessels et al. [25] prepared type III GBS conjugate vaccines by linking saccharides of average MWs, at 38, 105 and 349 kDa, respectively, to TT at multiple sites along the sugar chains. PS-specific IgG responses in mice correlated with the saccharide’s MW, and the protective efficacy induced was lower for the smaller saccharide conjugate. Michon et al. [26] conjugated different lengths of type II (in the range 15–51 kDa) and type III (in the range 11–41 kDa) GBS saccharides to TT using various chemistries, and reported that, for the type II conjugates, immunogenicity in mice increased with decreasing saccharide size, whereas for type III conjugates, the size of the OS did not significantly affect immunogenicity (Figure 1A). 

### 2.3. Streptococcus pneumoniae 

*S. pneumoniae* serotype 4 CPS (average MW 40–120 kDa) and OS (average MW 9.6 kDa) were randomly conjugated to TT [27]. In mice, CPS conjugates provided greater saccharide-specific IgG and IgM responses, both after primary and booster immunization, than the OS conjugates. Similar results were obtained in a subsequent study in human infants; when two pentavalent CPS-CRM_197_ vaccines (serotypes 6B, 14, 18C, 19F, and 23F) containing native CPS or corresponding OS were compared, PS conjugate vaccines were generally more immunogenic. The glycoconjugates were produced using random chemistry [28].

In another vaccine study (serotypes 3, 6A, 18C, 19F, and 23F, and pneumococcal common C-PS), OS at different lengths (3–14 kDa for serotype 3, 11–140 kDa for 6A, 6–15 kDa for 18C, 7.8–100 kDa for 19F, 8–16 kDa for 23F, and 1.3–10.4 kDa for C-PS) were coupled through the reducing end to TT and administered to rabbits. Chain length did not significantly affect immunogenicity, with the exception of serotype 19F, where increased saccharide length was associated with decreased immunogenicity [29]. Arndt and Porro [30], when testing *S. pneumoniae* OS serotypes 6A, 14, 19F, and 23F, terminally-linked to CRM_197_, found that shorter chain OS conjugates (average 3–6 RU of OS) gave a significantly higher antibody response in rabbits than longer chain OS conjugates (average 10–14 RU of OS), for all serotypes.

Conversely, when Lafarriere tested *S. pneumoniae*, 14 CPS fragments of different lengths (1.3 to 150 kDa), terminally linked to TT in rabbits [31], immunogenicity and opsonophagocytic activity increased with the increasing size of the saccharide. Interestingly, the predominant antibody response to *S. pneumoniae* 14 CPS appeared to target an extended conformational epitope. Pawloski et al. [32] compared *S. pneumoniae* 14 and 23F CPS glycoconjugates containing medium-sized (38–50 kDa) or small-sized (6 kDa) OS terminally linked to TT in rabbits. For serotype 14, the conjugate containing medium-sized OS induced higher antibody levels than the conjugate containing small-sized OS, whereas the 23F conjugates were similar, irrespective of the saccharide size.

When serotype 6A OS of three different lengths were coupled to TT [33], mice responded similarly to vaccines with 2 and 14 RU, and minimally to a conjugate with 7 RU, although interpretation is complicated, because the conjugates differed in their saccharide to protein ratios. In two-year-old children, the efficacy of the same conjugates increased with increasing chain length, thus demonstrating that animal studies do not necessarily translate into clinical findings. In accordance with the findings of this study, when two bivalent *S. pneumoniae* (serotype 6B and 23F) conjugate vaccines, with native CPS or 10–20 RU of OS terminally linked to CRM_197_, respectively, were compared in young children, the native CPS conjugates were significantly more immunogenic [34]. More recently, when *S. pneumoniae* serotype 3 OS were terminally conjugated to TT, pentasaccharide and hexasaccharide conjugates elicited significantly higher IgG antibodies than hepta- and octasaccharide conjugates in mice [35] (Figure 1B).

### 2.4. Neisseria meningitidis

When MenC OS of 2–4, 4–10, and 10–50 kDa, conjugated to P64k by reductive amination following saccharide oxidation, were tested in mice, the IgG antibody levels and bactericidal activity were not statistically different [36]. With a set of MenX OS of different length (average number of 5, 10 and 20 RU) conjugated to CRM197, the 20 RU OS conjugate induced in mice the optimal response in terms of both anti-MenX antibodies and SBA titers [37] (Figure 1C).

### 2.5. Salmonella typhi 

Szu et al. [38] compared the immunogenicity of conjugates made with *S.* Typhi Vi CPS (approximately 3 × 10^3^ kDa), or lower MW Vi OS (approximately 46 kDa), that were randomly coupled to a cholera toxin B subunit (CTB). The high MW PS conjugate elicited higher levels of Vi antibodies than the lower MW OS saccharide conjugate in both mice and rhesus monkeys. More recently, a Vi-CRM_197_ glycoconjugate vaccine was tested in humans that elicited a higher anti-Vi IgG antibody response than unconjugated Vi after a single dose, even when used at a lower dose (5 vs. 25 μg Vi); however, the half-life of Vi-specific antibody titers showed no differences after conjugation or PS vaccine administration, and a second dose of the conjugate vaccine had no incremental effect on antibody titers in either children or older infants [39,40]. It was speculated that, despite conjugation to the carrier protein, very long Vi chains may retain a strong ability to induce T-independent responses. To test this possibility, Vi-CRM_197_ conjugates made with saccharides (with average sizes of 9.5, 22.8, 42.7, 82.0, and 165 kDa) were prepared and tested in mice [41]. Long-chain-conjugated Vi (165 kDa) induced a response in both wild-type and T cell-deficient mice. In marked contrast, short-chain Vi (9.5 to 42.7 kDa) conjugates induced a response only in wild-type mice and not in T cell-deficient mice. Moreover, in neonatal mice, long-chain, but not short-chain Vi conjugates, induced the late apoptosis of Vi-specific B cells in the spleen and the early depletion of Vi-specific B cells in bone marrow, thus resulting in hyporesponsiveness and a lack of long-term persistence in terms of Vi-specific IgG in serum and IgG^+^ antibody-secreting cells in bone marrow. 

When full-length or fragmented Vi random glycoconjugates, bound to different carrier proteins (CRM_197_, Diphtheria Toxoid (DT), and Tetanus Toxoid (TT), were compared in mice, full-length conjugates elicited higher anti-Vi IgG responses after the first immunization; however, the difference became statistically insignificant after the second immunization [42] (Figure 1D).

### 2.6. Salmonella typhimurium 

When tetra-, octa-, and dodecasaccharides of *Salmonella* Typhimurium O-SP were conjugated through the terminal reducing ends to bovine serum albumin (BSA), anti-LPS antibody titers increased with OS size in both mice and rabbits [43]. In another work, Rondini et al. found that CRM_197_ selective conjugates made with *S.* Typhimurium O-SP at high MWs (average 71 RU) were considerably less immunogenic than glycoconjugates based on O-SP at medium MWs (average 25.5 RU) [44] (Figure 1D).

### 2.7. Francisella tularensis 

Using glycoconjugates of the *Francisella tularensis* O-SP, bearing different saccharide sizes coupled to TT, it was demonstrated that conjugates made using random chemistry, with the higher MW O-SP being derived from a genetically modified bacterium (around 220 kDa), elicited greater protection in mice against bacterial challenges than native MW (80 kDa) and low MW (25 kDa) preparations [45]. Interestingly, both the conjugates with genetically-enlarged O-SP and native size O-SP induced lower antigen-specific IgG titers than a low MW O-SP conjugate; however, the relative affinity of the antibodies induced by the glycoconjugates was directly correlated with the size of the O-SP used. This finding suggests that there is not always a direct correlation between the amount of IgG elicited by a glycoconjugate and the level of protection induced, thus encouraging a focus on the development of glycoconjugates inducing highly effective antibodies (Figure 1E). 

### 2.8. Shigella

The immunogenicity of *Shigella dysenteriae* type 1 and *Shigella sonnei* conjugates was significantly improved using lower MW saccharides compared with full length O-SP, and they were bound at their reducing ends to the protein carrier at defined densities. Pozsgay et al. [46], studying synthetic *S. dysenteriae* type 1 OS coupled to human serum albumin at multiple attachment points, found that conjugates with octa-, dodeca-, and hexadecasaccharide (2, 3 and 4 RU, respectively) elicited higher antibody levels than a conjugate made with full length O-SP (average 27 RU). Furthermore, *S. sonnei* conjugates bearing low MW O-SP fragments (average 3.5 RU), which were terminally linked to BSA or DT, induced significantly higher antibody levels in mice than the full-length O-SP (average 29 RU) conjugated to *r*EPA by random chemistry [47].

Phalipon et al. [48] found that a synthetic sequence with three RU of the *Shigella flexneri* 2a O-SP, conjugated to TT, showed higher immunogenicity and better protective efficacy in mice than conjugates with one or two RU of the saccharide antigen, thus suggesting that a minimum number of RU may be necessary to induce an optimal immune response. In accordance with this finding, a conjugate with one RU did not react with anti-*S. flexneri *2a serum by immunodiffusion [49]. In contrast, for *S. dysenteriae* type 1 conjugates, even O-SP with one RU coupled to BSA reacted with anti *S. dysenteriae* type 1 serum by immunodiffusion and was immunogenic in mice [49]; however, for *S. flexneri* 6, glycoconjugates with O-SP, with an average of one or two RU conjugated to BSA, only induced low antibody levels in mice, whereas those with an average of seven RU or full-length O-SP induced higher antibody levels. When Raso et al. [50] compared *S. flexneri* 6 O-SP of different lengths (174, 22 and 1.7 kDa), conjugated to CRM_197_, in wild type and T-cell deficient mice, O-SP length was found to have no major impact on the immune response that was independent from the conjugation chemistry used; indeed, only the high MW conjugate induced an anti-O-SP IgG response that was significantly different from background levels in T-cell deficient mice (Figure 1F).

### 2.9. Vibrio cholerae 

When synthetic hexa-, octa-, and decasaccharides of the *Vibrio cholerae* O1 serotype Ogawa O-SP were terminally conjugated to TT, decasaccharide conjugates elicited the highest anti-LPS IgG [51] (Figure 1G).

### 2.10. Haemophilus influenzae Type B 

In a study of rats which used end-linked Hib PS-TT conjugates of various sizes (10, 50, and 100 kDa) there was a trend toward higher immunogenicity with reduced saccharide size, despite a lack of statistical significance [52]. When Hib OS with an average of 8 or 20 RU were coupled by selective chemistry to DT, the conjugates were equally immunogenic in rabbits and human adults; however, the 20-mer vaccine was significantly more immunogenic than the 8-mer vaccine in human infants [53], thus suggesting that the importance of vaccine structure and saccharide size may be age-dependent and related to the development status of the immune system. A subsequent study compared the OS mean lengths (4, 6, or 12 RU) with different terminal residues, and they were terminally conjugated to CRM_197_ in adults and infants. This study found that the OS chain length and terminal residue structure were less critical variables for the immune response than the extent of OS loading. This result also suggests that an extended epitope is not required in the generation of a protective immune response by polyribosylribitol phosphate (PRP) [54].

Peeters et al. [55] showed that a synthetic tetramer of Hib CPS, conjugated to either DT or TT, induced antibody levels in adult mice and non-human primates that were higher than those induced by a synthetic OS trimer glycoconjugate, thus suggesting a minimum of four RU for an optimal immunological response to Hib conjugate vaccines. Adult monkeys responded equally well to the tetrameric OS-TT conjugate and to an OS-CRM_197_ conjugate of 20 RU, although the conjugates used different carrier proteins. When Chong et al. [56] tested synthetic Hib OS dimers and trimers coupled to TT in rabbits, the trimer was more immunogenic; however, the synthetic Hib trimer-TT conjugate did not elicit antibody responses in mice and guinea pigs. 

To summarize, the nature of the antigen influences the effect that saccharide chain length can have on immunogenicity. Furthermore, the impact of saccharide length also appears to be affected by other interdependent factors, including saccharide loading, conjugation chemistry, and the carrier protein. Some efforts have been made to produce sets of glycoconjugates that differ only in saccharide chain length, with similar saccharide loading or conjugate sizes. Conclusions concerning the effect of saccharide chain length are saccharide-specific and can be conflicting; however, it is generally recognized that a minimum saccharide chain length is necessary to induce immune responses, and this threshold value is strongly hapten-specific. In addition, since most of the published data on the influence of sugar chain length comes from studies in mice or rabbits, it is necessary to extend the observation to human studies to better assess the importance of this variable in clinical settings (Figure 1H). 

## 3. Methods to Reduce Polysaccharide Size

Several methods can be adopted to reduce the PS size, but not all are necessarily suitable for a specific PS structure, nor can they be scaled in an industrial process (Table 1). In general, fragmentation methodologies have a different degree of specificity, with some techniques capable of targeting well-defined chemical bonds in PS structures (for example deamination, and oxidation with sodium periodate). Moreover, other random methods, such as acidic and basic hydrolysis, can affect different types of chemical bonds. Depending on the specific PS and fragmentation conditions, methods to reduce PS size (in particular, random approaches) may result in OS with larger polydispersity compared with the original PS; therefore, it is important to monitor the fragmentation process by using in-process controls to stop the reaction at a predetermined average chain length, and to apply size-exclusion chromatography or alternative methods to control polydispersity [20]. The chemical structure of the PS RU discussed in this review are reported in Figure 1. 

### 3.1. Hydrolysis

**Scheme 1:**
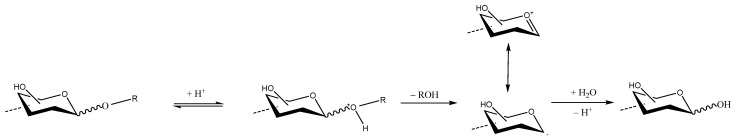
Acidic hydrolysis mechanism of a glycosidic bond. The reaction mechanism behind the acidic hydrolysis of glycosidic bonds is complex. In principle, both the glycosidic O atom and the ring O atom can be protonated. The protonation of the glycosidic O atom is reversible and fast. The rate-limiting step is probably the cleavage of the glycosidic linkage which forms an anomeric carbocation with an energetically unfavorable conformation. The carbocation can finally react with water to give a mixture of α,β-anomers with the original ring form; however, conversions of the pyranose/furanose ring via protonation of the ring O atom in an earlier stage, and/or the mutarotation of the original released ring form, will eventually produce a mixture of α,β-pyranose and α,β-furanose ring forms (not shown) [58].

The glycosidic bond of PS—the covalent bond that joins the hemiacetal group of a monomer with the hydroxyl group of the adjacent sugar—is generally much more resistant to basic hydrolysis than to acid hydrolysis. For this reason, basic solutions are often used to extract bacterial or cell wall PSs, whereas acidic solutions are avoided. During acidic hydrolysis, one molecule of water is consumed for every glycosidic linkage cleaved (Scheme 1). Strong acidic hydrolysis (such as with sulfuric acid (H_2_SO_4_) and trifluoracetic acid (TFA) solutions), often temperature-aided, is also used when monosaccharide generation is required for PS quantitative and/or qualitative analysis; however, not all glycosidic linkages are cleaved at the same rate, and the conditions for partial hydrolysis with minimal destruction of the constituents have to be determined experimentally. For example, the glycosidic linkages of furanosyl rings and deoxy sugars can generally be readily hydrolyzed with an acidic solution, and the hydrolysis of 6-deoxyhexoses with glycosidic linkages is about five times faster than their corresponding hexoses [57]. In contrast, the glycosidic bonds of uronic acids, and 2-amino-2-deoxyhexose, are particularly resistant to acid hydrolysis. If a PS contains only a limited number of acid-labile glycosidic linkages, a partial hydrolysis will produce a mixture of monosaccharides and OS, and their characterizations can provide important information on the structure of the PS. 

### 3.2. Mild Acidic Hydrolysis

Chemical hydrolysis has been used successfully in some conjugate vaccine development programs (e.g., [16,59,60,61]). Although the glycosidic bonds of a PS may present different susceptibilities to hydrolysis, the fragmentation process can be a random event, with the sugar polymer breaking down at virtually any point along the chain, thus increasing the polydispersity of the sample, causing it to require further sizing and purification steps. Several methods have been introduced to monitor PS fragmentation, including the use of optical activity as a physical property correlating with the degree of polymerization for Hib PS [60]; however, undesired side reactions may be associated with chemical hydrolysis impacting side-chain structures (e.g., impact of bases on O-acetyls of *S*. Typhi Vi PSs [62], or the impact of acids on *F. tularensis* O-SP [45]), which modify the antigenicity of the immunogen. The presence of a single acid-labile linkage in the RU of Hib (Rib-P) and Men (ManNAc-P (MenA) and NeuNAc (MenC, Y and W-135)) CPS allows the use of mild acid hydrolysis (which has little impact on O-acetylation) for side-reduction purposes [20] (Table 1).

#### 3.2.1. *Neisseria meningitidis*

The presence of a single acid-labile linkage in the RU of Men CPS (Figure 1C) allows the use of mild hydrolysis to generate active OS for conjugation [63]. Indeed, preparation of MenACWY-CRM_197_ by GSK (technology first developed by Novartis Vaccines) [64] involves purification, mild acidic hydrolysis using temperature-aided acetic acid solutions—with the protocol varying based on the specific sugar—and size fractionation of the OS chains, which results in a population profile of OS of restricted sizes. For example, MenC CPS can be hydrolyzed at 86 °C in a 10 mM sodium acetate buffer of pH 5 at a PS concentration of 10 mg/mL for about 4 h to produce OS that can be separated by anion exchange chromatography [60]. This process eliminates very short chains, which are thought to be less immunogenic, and selects intermediate chain length OS that are useful for an efficient and consistent conjugation process [59]. The reducing end ketone is then converted to an amino group by reductive amination with aqueous ammonium chloride, which is further functionalized by adding an N-hydroxysuccinimide diester of adipic acid that is conjugated to CRM_197_ amino groups [63]. Mild acidic hydrolysis with 50 mM NaOAc was also used to produce OS from Men X CPS, with the aim of defining the structural and minimal protective epitope recognized by functional antibodies. Different conditions (temperature and reaction times) were used depending on the OS MW target. For example, to produce MenX OS with an average of 5 RU, the hydrolysis was performed in 50 mM NaOAc, with a saccharide concentration of 2.5 mg/mL at pH 4.0, 80 °C for ∼18 h, and two times overnight at RT [37].

#### 3.2.2. *Hemophilus influenzae* Type B 

A technology similar to the one reported above for the acid-catalyzed hydrolysis of Men CPS has been applied by GSK to produce Hib conjugate vaccines [65]. A 10 mg/mL solution of Hib CPS (Figure 1H) in 0.01 M acetic acid was heated at 70–80 °C until the OS generated had an average polymerization degree of 10 RU [60]. Conjugation to CRM_197_ was performed following a similar procedure to that reported above for MenC OS. In addition, Hib CPS acidic hydrolysis was applied to study the impact of PS chain length, terminal group, and PS/protein ratio in conjugate vaccines using CRM_197_ as carrier protein [54]. Oligomers of PRP—the RU of Hib—with ribose at the reducing end and ribitol as the terminal group, were obtained at pH 3 with 4, 6, and 12 RU, and their conjugates were prepared by direct reductive amination. In addition, the hydrolysis of PRP calcium salt at pH 1, which mainly affected phosphate diester groups, allowed the production of oligomers with monophosphate at the nonreducing end. These oligomers were used to produce a conjugate vaccine based on a 7 RU OS that was then treated with periodate to oxidate the terminal ribitol before conjugation to CRM_197_ by reductive amination.

#### 3.2.3. *Streptococcus pneumoniae*

Acidic hydrolysis has been also used to generate *S. pneumoniae* OS. Partial acidic hydrolysis of the serotype 3 CPS (Figure 1B) was performed by heating a 0.3 M trifluoroacetic saccharide solution for 3 h at 100 °C. The OS mixture was then purified by ion-exchange chromatography, obtaining fragments containing one to seven (→3)-β-d-GlcpA-(1→4)-β-d-Glcp-(1→) RU [66]. A similar methodology was applied for the evaluation of the immune responses of mice and two-year-old children using *S. pneumoniae* type 6A–protein conjugate vaccines of differing saccharide chain lengths (Figure 1B). OS of 2, 7, and 14 RU lengths were obtained by hydrolyzing the PSs at 10 mg/mL in 0.1 M acetic acid at 100 °C for 1.5–11 h (depending on the MW target) [33]. 

### 3.3. Thermal Hydrolysis

Thermal hydrolysis can occur at temperatures above 100 °C and it does not require the presence of acids. This approach, though it comes with some challenges, could be made scalable and reproducible, but it is not straightforward, as high temperatures may also lead to the degradation of the PS structure in addition to fragmentation. The most thermally labile saccharide–saccharide bond is the main target; however, side reactions can occur, thus making this methodology unsuitable for certain saccharides (e.g., PSs with side chains containing sialic acid moieties) (Table 1).

#### 3.3.1. *Hemophilus influenzae* Type B

Sanofi Pasteur has developed a methodology for making conjugates where the saccharide component is first thermally hydrolyzed to produce shorter saccharide chains before activation and conjugation [12]. Heating the Hib CPS (Figure 1H) in water for 15 min at 100 °C is generally enough to guarantee that less than 20% of the molecules are of a molecular size smaller than 200 kDa, and less than 20% of a molecular size greater than 2000 kDa. The saccharide chains are then activated using cyanogen bromide (CNBr) and coupled to adipic dihydrazide (ADH) derivatised tetanus toxoid (TT) through a covalent isourea linkage [12].

### 3.4. Reactive Oxygen and Nitrogen Species

Reactive oxygen species (ROS) are defined as chemically active products generated by partial oxygen reduction, whereas reactive nitrogen species (RNS) refer to reactive products derived from reactions with nitric oxide (NO) [67]. ROS/RNS can be classified as free radicals, which contain one unpaired electron, or nonradicals. The former generally includes a superoxide ion radical (O_2_^−^), hydroxyl radical (^•^OH), peroxyl (ROO^•^), alkoxyl radicals (RO), and NO radical (NO^•^). The latter includes hydrogen peroxide (H_2_O_2_), organic peroxide (ROOR′), ozone (O_3_), hypochlorous acid (HClO), singlet oxygen (^1^O_2_), aldehydes (HCOR), and peroxynitrite (ONOOH) [68]. Several ROS/RNS can individually or concertedly cause the backbone scission of a PS, thus generating smaller fragments [67]. Here, we only report the evidence and mechanisms related to PS fragmentation methods that have been used in the context of glycoconjugate vaccines. Although the different approaches proposed have distinct targets and properties, they are all characterized by simplicity, scalability, and reproducibility (Table 1). 

### 3.5. Hydrogen Peroxide 

**Scheme 2:**
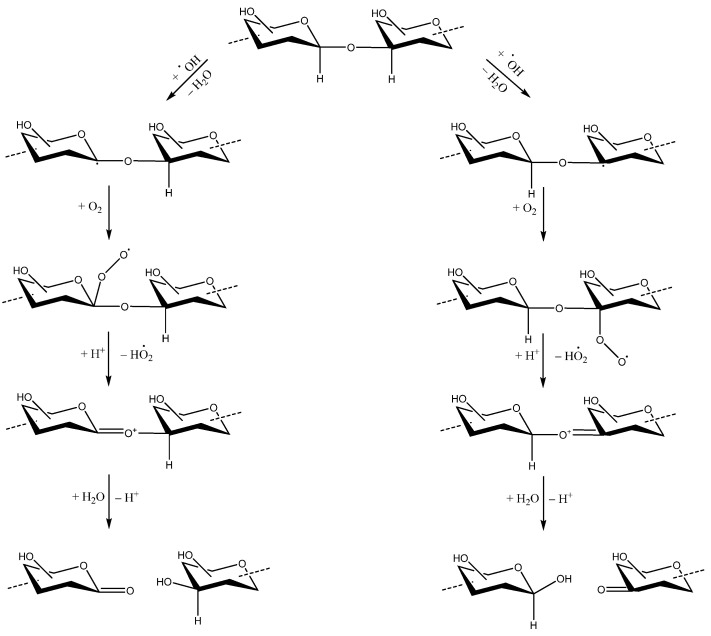
Proposed reaction mechanism of ^•^OH on a PS chain. The diagram shows the predicted reaction that occurs after ^•^OH subtracts a hydrogen atom from the C–H of carbons involved in the glycosidic bond, thus resulting in fragmentation of the PS [75].

PS fragmentation using H_2_O_2_ has often been used to degrade PSs, including those of bacterial origin [69]. This is mainly related to its mild reaction conditions, high efficiency, inability to alter the saccharide structural integrity, and ease of ^•^OH radical formation [69]. The hydroxyl radical can remove hydrogen atoms at all ring C–H bonds of aldoses, uronic acids, and other sites on carbohydrates, except C-2 of N-acetyl hexosamine [70,71]. The removal of the hydrogen atom generates carbon-center radicals. The carbon radicals forming glycosidic bonds will undergo a β-scission reaction with a consequent breakdown of PS chains [70,71,72] (Scheme 2). The activation of H_2_O_2_ is necessary to produce reactive ^•^OH radicals that can effectively depolymerize the PS; this can be achieved using several methods (e.g., with UV, ascorbic acid, Fe^2+^/ascorbic acid, ultrasound, or irradiation). Generally, by using only H_2_O_2_, or H_2_O_2_ in combination with other degradation techniques, the main chain structure in the degraded or modified PS may not be altered, but the side chain structure can change [73]. Hydrogen peroxide has been used to generate OS for *Neisseria meningitidis* [74] and *S.* Typhi (Vi antigen) [41,42] in order to produce glycoconjugate vaccines.

#### 3.5.1. *Neisseria meningitidis*

Sanofi Pasteur developed a methodology for sizing CPS from MenA, C, W-135, and Y (Figure 1C) based on hydrogen peroxide treatment [74]. The Men CPS are partially fragmented by heating a 1.25 mg/mL PS solution in a 50 mM sodium acetate buffer at pH 6.0 at 55 °C with 1% hydrogen peroxide for the time necessary for each specific serogroup CPS to reduce the native size from 500–1500 kDa to less than 100 kDa (preferably between 12–25 kDa). Interestingly, the characterization of both the reducing end group and the repeating sialic acid units of fragmented MenC CPS by Liquid Chromatography/Mass Spectrometry methodology shows that fragmentation occurs by cleavage at the α2-9 glycosidic linkage. This yields intact sialic acid units without the loss of O-acetyl or carboxyl groups, and it results in a higher concentration of aldehyde groups at the reducing end terminus due to an increase of shorter saccharide chains in the solution [76]. The Men OS are then activated with adipic acid dihydrazide (ADH) by applying carbodiimide crosslinker chemistry (targeting the COOH group on the RU) and/or by reductive amination with sodium cyanoborohydride (NaBH_3_CN). This reduces the Schiff’s base, generated between the aldehyde unit in the reducing end and ADH. Once activated, the PSs are conjugated to carrier proteins using carbodiimide chemistry.

#### 3.5.2. *Salmonella typhi*

Hydrogen peroxide has been also used to reduce the size of the very stable *S.* Typhi Vi CPS [41,42]. Vi CPS, freeze dried as sodium salt, was solubilized in water, and H_2_O_2_ was added to give a final concentration of 2.5 mg/mL Vi and 5% (*wt*/*v*) H_2_O_2_ in water. The mixture was heated at 80 ± 0.5 °C for 2 h. Populations of different average sizes were obtained and separated by anion exchange chromatography (Figure 1D). 

### 3.6. Ozone

**Scheme 3:**
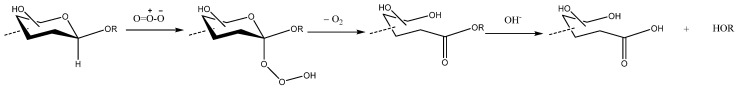
The ozonolytic cleavage of β-d-glycosidic bonds [77].

The fragmentation of PSs by ozone in an aqueous solution is believed to occur via three main mechanisms [77]: (1) selective ozonolytic oxidation of β-d-aldosidic linkages; (2) nonselective oxidative degradation by radical species; and (3) nonselective acid hydrolysis. Among these, the first is the predominant reaction, and it leads to the saponification of newly formed aldonic acid esters in order to yield shorter saccharides (Scheme 3). It has been highlighted that the ozonolytic oxidation of aldosides proceeds under stereo electronic control, and that it prefers the aglycone conformation, in which each oxygen has one of its lone-pair orbitals antiperiplanar to the alkylidene C–H bond. For this reason, glycosidic bonds with different conformations can have different kinetic reactions with ozone, thus allowing the selectivity in the cleavage of the β-d-linkages of PSs [77].

#### 3.6.1. Group B *Streptococcus*

The different types of GBS CPS (Figure 1A) contain a backbone with β-d-linkages—the most susceptible to ozone treatment—but their selectivity is hampered by the fact that there is more than one per RU. Ozonolysis can be carried out in either an aqueous solution [78] or in an organic solvent following acetylation of the PS [77]. Ozonolysis was used to design conjugate vaccines with size-specific OS antigens and size-controlled coupling. After peracetylation, type III CPS was dissolved in ethyl acetate (2 mg/mL) and aerated using a stream of ozone/air gas (21% O_3_, 3.17 mL/s) at room temperature. The reaction was terminated when the products reached the desired molecular size by stopping the addition of ozone (e.g., starting with a 61-kDa type III GBS PS, 1 h of ozonolysis was required to decrease the average molecular size to 20 kDa, and 10 h to 10 kDa) [77]. The product was then functionalized with ethylenediamine, and then with a squarate linker, before conjugation with a carrier protein [77,78,79].

### 3.7. Nitric Oxide and Nitrous Acid

**Scheme 4:**
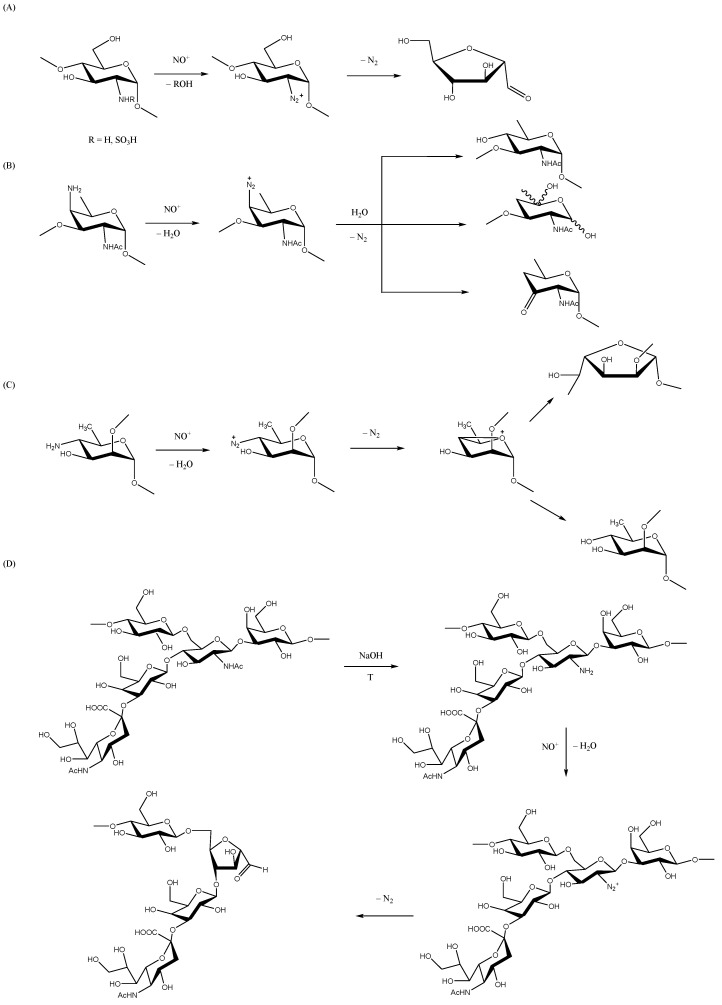
The deamination of 2-amino or 4-amino hexamine residues: 2-amino-2-deoxy sugars (**A**), 2-acetamido-4-amino-2,4,6-trideoxygalactopyranosyl (AATp) (**B**), 4-amino-4,6-deoxymannopyranosyl residues (**C**) (partially adapted from [67]), GBSIII CPS (**D**) (only the reaction leading to fragmentation of the PS is shown, but the N-acetyl moiety of the sialic acid may react as well).

Nitric oxide (NO) and nitrous acid (HNO_2_) share a common intermediate, the nitrosonium cation (NO^+^), which is capable of nitrosating free amino or N-sulfo groups (but not N-acetyl groups). The nitrosation reaction requires an acidic environment (pH = 4 or pH = 1.5 for amino or N-sulfo groups, respectively). In the case of 2-amino-2-deoxy sugars, the reaction produces a loss of nitrogen with a ring contraction to generate 2,5-anhydro sugars coupled with the elimination of the aglycone (Scheme 4A). Moreover, 4-amino-4-deoxy sugars are also susceptible to deamination as a result of a similar mechanism [80,81]. Not all deamination reactions lead to breaking glycosidic bonds, as this depends on the favored conformation of the 4-amino group and the glycosidic linkage. As such, the deamination of 4-amino groups of 3-linked 2-acetamido-4-amino-2,4,6-trideoxygalactopyranosyl (AATp) residues will break both C-3 and C-1 of AATp (Scheme 4B; [80]), thus resulting in PS fragmentation, whereas deamination of 4-amino groups of 2-linked 4-amino-4,6-deoxymannopyranosyl residues will not break the glycosidic bond (Scheme 4C) [81]. 

#### 3.7.1. Group B *Streptococcus*

The nitrosation reaction has been used to fragment type II and III GBS CPS (Figure 1A) to generate size-reduced OS that bear a terminal aldehyde unit for conjugation purposes [26]. First, the N-acetyl-glucosamine moieties of the RU are partially de-N-acetylated via heating in an alkaline solution. Then, nitrous acid treatment leads to saccharide fragmentation with the generation of a 2,5-anhydro-d-mannose residue, which contains a terminal aldehyde group (Scheme 4D). Importantly, this procedure has been used to generate type III GBS OS, thus enabling a study of their interactions with functional monoclonal antibodies at the crystalline structure level [82]. Although as a side reaction, this procedure may lead to the partial de-N-acetylation of the sialic acid residues, this issue could be circumvented by using a sialyltransferase to introduce terminal sialic acid residues prior to nitrous acid treatment [83].

#### 3.7.2. *Streptococcus pneumoniae*

A similar approach was used for preparing *S. pneumoniae* 14 OS fragments of different lengths for conjugation to TT. Serotype 14 CPS (Figure 1B) was first partially de-N-acetylated and then treated with nitrous acid to yield saccharide fragments (1.4 to 150.0 kDa) that have a free aldehyde at the reducing end. The OS were then conjugated to TT via reductive amination [31]. 

### 3.8. Periodate Oxidation

**Scheme 5:**
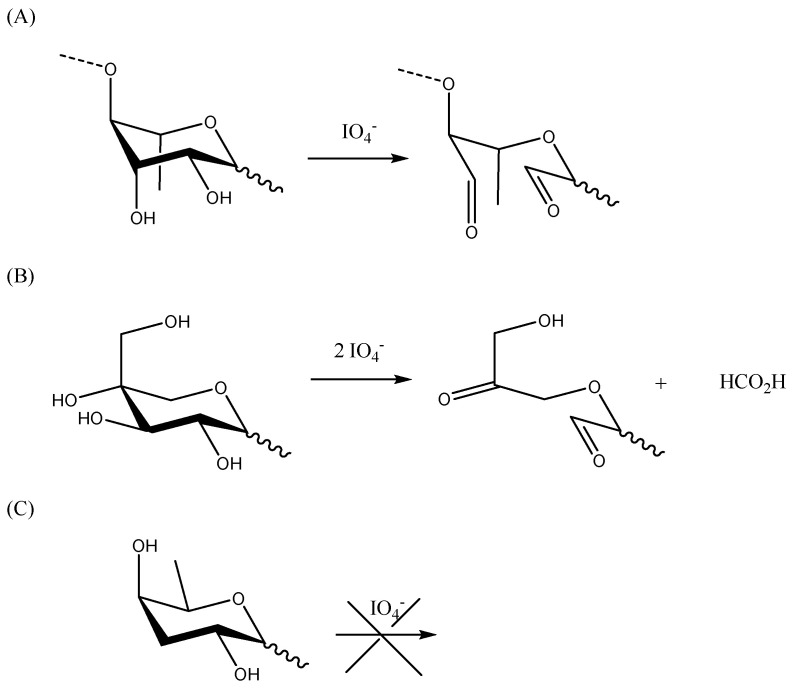
Periodate oxidation of: (**A**) sugar unit forming two aldehydic groups with the cleavage of one carbon chain (e.g., 1,4-linked rhamnose); and (**B**) sugar unit forming two aldehydic groups with the cleavage of two carbon chains (e.g., terminal glucose). (**C**) Sugar units that do not have adjacent hydroxyl groups do not undergo periodate oxidation (e.g., terminal abequose) [88].

Vicinyl-glycols (two neighboring hydroxyl groups) of PS can react with periodic acid or its salts (e.g., sodium periodate) to generate two aldehydic groups upon the cleavage of the carbon chain. This reaction will quantitatively consume one molar equivalent of periodate. Nonterminal units, such as 1,2- or 1,4-linked residues, will consume one equivalent of periodate without formation of formic acid (Scheme 5A). In the case of αβγ-triols, a double cleavage of the carbon chain will occur on both sides of the β position. This reaction consumes two molar equivalents of periodate and forms two aldehydic groups and one formic acid (Scheme 5B). Sugar units that do not have adjacent hydroxyl groups, including 1,3-linked residues or branched at C-2 or C-4 positions, will not be affected by this reaction (Scheme 5C). Due to this different selectivity, the oxidation reaction can also be used to elucidate the structural information of PS.

The amount of sodium periodate used for oxidation is an important parameter for controlling selectivity of the process. Sodium periodate, at a concentration of 1 mM at nearly 0 °C, specifically cleaves only at the adjacent hydroxyls between carbon atoms 7, 8, and 9 of sialic acid residues [84,85]; however, the oxidation of PSs, using sodium periodate at 10 mM or greater, at room temperature, will also result in the cleavage of other adjacent hydroxyl-containing carbon–carbon bonds in other sugars [86]. 

Periodate oxidation is likely to be the simplest route for transforming the hydroxyl groups of saccharides into amine-reactive aldehydes for further conjugation or functionalization. As a rule, *cis*-glycols react faster than *trans*-glycols, and there is evidence for the formation of heterocyclic reaction intermediates, although their formation is not necessary for a reaction to occur. The cyclic ester is formed more readily for cis diols than for trans diols; therefore, the cleavage rate of cis diols is faster than the cleavage rate of trans diols [87]. When applicable, periodate oxidation has often been used for conjugate vaccines due to its simplicity, and there is a possibility of selectively oxidizing different moieties of the sugar by modulating the oxidant concentration (Table 1).

#### 3.8.1. *Neisseria meningitidis*

This methodology is used for the preparation of the licensed Nuron Biotech Meningitec^®^ and Pfizer NeisVac-C^®^ MenC conjugate vaccines. For Meningitec^®^, the controlled treatment of MenC CPS (Figure 1C) with sodium periodate generates two aldehydes in the non-O-acetylated RU →9)-α-d-Neu5Ac-(2→ (5–10% randomly distributed), and it oxidizes and cleaves the C7-C8 bond to generate shorter saccharide chains. The aldehyde groups are then conjugated to the amino groups of CRM_197_ by reductive amination. NeisVac-C^®^ is different in that MenC CPS is de-O-acetylated using NaOH 0.1 M before fragmentation/activation with NaIO_4_, and conjugation to TT occurs via reductive amination [89].

#### 3.8.2. *Hemophilus influenzae* Type B

The Wyeth Lederle Hib conjugate vaccine has been produced using a method based on an aldehyde-reductive amination reaction [53,90], which was previously used in the synthesis of monovalent meningococcal conjugates [91]. Given the chemical structure of Hib (Figure 1H), periodate oxidation led to the fragmentation of PRP with the spontaneous generation of two terminal aldehydes per saccharide. The degree of fragmentation can be controlled by the amount of periodate used, and the activated saccharide directly conjugated to CRM_197_ by reductive amination generates a partial crosslinked conjugate vaccine (with the sugar able to react with the carrier protein at both the terminal units) [53,90,91].

#### 3.8.3. *Streptococcus pneumoniae*

*S. pneumoniae* 4 OS were obtained by treating serotype 4 CPS (Figure 1B) with periodate (35 mM IO_4_^−^, 75 h at 4 °C), and the immunogenicity of the corresponding glycoconjugates was obtained by coupling to TT via EDC chemistry, and compared with the full size PS glycoconjugates in mice [27]. 

### 3.9. Enzymatic Hydrolysis

**Scheme 6:**
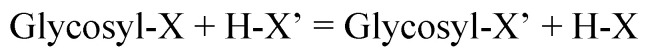
Enzyme-catalyzed glycoside cleavages (and syntheses). X = saccharide, 1,2-glycal, phosphate residue, nucleoside diphosphate, polyprenol diphosphate. X’ = OH or OR (including saccharides).

Glycoside-cleaving enzymes are a heterogenous group, which, in a somewhat selective way, hydrolyze the wide variety of O-glycosidic linkages found in glycosides, oligo- and polysaccharides, and glycoconjugates. Enzyme-catalyzed glycoside cleavages (and syntheses) are considered variants of the same reaction (Scheme 6). They are catalyzed by a single type of enzyme, a glycosylase, a group which includes glycoside hydrolases, glycosyl transferases, phosphorylases, and some lyases [92]. This equation describes carbohydrase reactions as an exchange between a glycosyl group and a proton at the X and X’ sites in the substrates.

Alternatively, glycoside-cleaving enzymes can also be categorized based on the action pattern, namely, (1) endoenzymes, which randomly cleave a molecule of a substrate into two smaller molecules, and (2) exoenzymes, which detach a monomer or a dimer at the nonreducing end of the substrate molecule. The synergistic action of both types of enzymes is not only significant in terms of the biological degradation of PSs using living cells, but it is also important for the industrial saccharification of PS resources, such as starch and cellulose; however, since enzymes only hydrolyze specific substrates, this method is not generalizable. Furthermore, some enzymes have been shown to preferentially hydrolyze smaller saccharides, which results in low yields of saccharide fragments of an adequate size to stimulate an immune response [24] (Table 1).

#### 3.9.1. Group B *Streptococcus*

Enzymatic degradation with endo-β-galactosidase from *Citrobacter freundii* has been proposed to produce heterogeneous pools of OS, starting from type III GBS CPS (Figure 1A) by cleavage of the β-d-Galp-(1→4)-β-d-Glcp linkage of the backbone [23]. Size exclusion chromatography was then used for the isolation of more homogeneous OS in the range of 1–5 RU [93]. Different from the previously discussed fragmentation method of GBS with nitrous acid treatment [26], this approach does not have the drawback of partially de-N-acetylating sialic acid residues, and this could be applied to all the GBS CPS types; however, the application of this method has been very limited due to the fact that the enzyme preferentially hydrolyzes smaller saccharides [24].

#### 3.9.2. *Salmonella typhimurium*

A promising approach for the preparation of OS involves bacteriophages, which can produce enzymes that are able to degrade PSs in order to gain access to the bacterial cell surface [92]. Enzymatic degradation with phage 36 endo-rhamnosidase of partially delipidated *S.* Typhimurium O-SP (Figure 1D) was used for the production of OS with 4, 8 or 12 RU, which were then conjugated through the terminal reducing ends to BSA, and the immunogenicity induced by the corresponding conjugate vaccines was compared in mice [43]. 

#### 3.9.3. *Klebsiella pneumoniae*

In a recent study, two PS depolymerases, identified from phages, were used to specifically cleave K1 and K2 CPS (Figure 1I)—the predominant *Klebsiella pneumoniae* CPS associated with invasive infections—into OS with intact structures. The obtained K1 and K2 OS were then separately conjugated to a CRM_197_ carrier protein to generate CPS-conjugated vaccine candidates that would be able to protect mice from subsequent infection with *Klebsiella pneumoniae* K1 and K2 serotypes [94].

#### 3.9.4. *Hemophilus influenzae* Type b

Acid phosphatase treatment of a conjugate vaccine based on a 7 RU OS—generated by the acidic hydrolysis of Hib CPS (Figure 1 H), followed by activation via periodate and conjugation to CRM_197_—was employed to produce a glycoconjugate that has ribose at the nonreducing end. This was used to understand that the terminal residue structure is not an important variable in the immune response generated by Hib conjugate vaccines [54].

### 3.10. Homogenization

Mechanical sizing using high pressure homogenization is one of the most widely used methods to produce glycoconjugate vaccines [20]. High shear rates are applied by pumping the process stream through a flow path with the desired dimensions. The high-pressure homogenization process is particularly suitable for reducing the size of PS containing non-saccharide substituents, such as O-acetyl, glycerol phosphate, pyruvyl groups, and so on, that are commonly present in pneumococcal, meningococcal, staphylococcal, and group B *Streptococcus* CPS [20]. Homogenization is a desirable approach for size-reduction, as once the PS concentration, pressure, and number of cycles are determined, the process can be highly consistent. Temperature control is essential to minimize undesired side reactions. This method reduces the polydispersity of PS molecules in a solution given that only larger—and not smaller—molecules are reduced in size; however, homogenization does have its limits, as the pressures needed to create very small PSs or OS may not be feasible. Each PS has to be evaluated independently to ensure that no adverse changes in the composition and structure of the RU occur [20] (Table 1).

### 3.11. Sonication

Similarly to homogenization, sonication reduces size via cavitation energy (expansion and collapse of micro-bubbles in the solution). It works by inputting energy directly into the solution via a probe, resulting in the physical shearing of the PS at equidistant nodes on the molecule, with inter-node distance being inversely proportional to energy input. As with homogenization, temperature plays a critical role in controlling the outcome of the reaction and the final saccharide polydispersity; however, sonication is quite difficult to conduct consistently as the efficiency of the probe changes with use, and the sonication energy is position-dependent, being highest at the point of the probe. This can result in increased polydispersity and inconsistent size reduction. Scalability is also an issue as the energy-to-volume translation is not exact. Additionally, the sonication probe is degraded during the sonication process, which means that metal contaminants could potentially be introduced into the PS solution [20]. Among other examples, sonication has been used to size-control *S. pneumoniae* CPS [95] (Figure 1B), *S*. Typhi Vi CPS [38] (Figure 1D), Hib CPS [96] (Figure 1H), *Staphylococcus aureus* Type 5 [97] and 8 CPS [98] (Figure 1L), and *Cryptococcus neoformans* serotype A glucuronoxylomannan (GXM) CPS [99] (Figure 1M) for conjugate vaccines (Table 1).

## 4. Bioconjugate Vaccines and New Ways to Modulate PS Length 

Bioconjugates are expressed directly in *E. coli* strains, and thus, they avoid the need to produce PSs and protein intermediates and perform conjugation reactions [100]. Glycoconjugate generation in *E. coli* requires the presence of genome clusters encoding bacterial PSs and a plasmid encoding the carrier protein, the latter of which has been genetically modified to include glycosylation sites in specific sequence patterns. Protein glycosylation is either oligosaccharyltransferase (OTase)-dependent (N-linked, when glycans are attached to the amide nitrogen of asparagine residues, or O-linked, when glycans are attached to the hydroxyl group of serine or threonine residues) or OTase-independent [101,102,103,104]. In the first case, glycans are moved *en bloc* from preassembled lipid-bound precursors to proteins in the periplasm. In the second case, glycosyltransferases displace monosaccharides from nucleotide-activated precursors to sequentially form glycoproteins in the cytoplasm. OTase-dependent strategies are currently applied to a greater extent, often using the N-linked OTase PglB from *Campylobacter jejuni* [105]. Strategies for controlling the saccharide chain length of bioconjugates are important. To the best of our knowledge, no specific studies have been conducted to compare the impact of sugar length in bioconjugate vaccines.

However, many of the bacterial pathways that regulate PS chain length are well-known [106], and have recently been modified and interchanged across species to generate saccharides of the desired length [45,107,108]. A mutant of the *F. tularensis* live vaccine strain with a significantly increased O-SP size (220 kDa vs. 80 kDa) was recently described, which had been generated by expressing a heterologous chain-length regulator gene (wzz) in *F. tularensis* from the related species *Francisella novicida*. The O-SP was then extracted and used to generate a protective conjugate vaccine against intranasal challenges with *F. tularensis*. 

In another work, the O-SP chain length regulators, *wzzB* and *fepE*, from *S.* Typhimurium I77 and *wzz2*, from *Pseudomonas aeruginosa* PAO1, were cloned and expressed in the homologous organism with a significant increase in the proportion of long or very long O-SP chains. A similar phenotype was obtained by overexpressing *wzzB* in *Salmonella* Paratyphi A and *S. flexneri*, and *wzz2* was also overexpressed in two other strains of *P. aeruginosa* [107].

Moreover, outer membrane vesicles (OMVs) from engineered bacteria, such as Generalized Modules for Membrane Antigens (GMMA), have been proposed as alternative delivery systems for O-SP, with the possibility of regulating sugar chain length [50,109]. Although they are not glycoconjugate vaccine technologies, they still represent an alternative system for delivering saccharide antigens. For *S. sonnei*, *S. flexneri* 2a, *S. flexneri* 6 and *S.* Typhimurium GMMA, it was found that the length of the O-SP (in the range 2.3–224 kDa for *S. sonnei*, 14–59 kDa for *S. flexneri* 2a, 1.7–174 kDa for *S. flexneri* 6, and 2.1–70 kDa for *S.* Typhimurium) does not play an important role in the elicited humoral response; however, when the *S. flexneri* 2a GMMA were mutated to display O-SPs mainly composed of only one RU, they did not elicit a significant anti-O-SP IgG response in mice, which is in agreement with previous studies that used more traditional glycoconjugates [49]. OMVs and GMMA can also act as carriers for heterologous PSs through chemical linkage [110,111,112]. Using GMMA as carriers for *S.* Typhi Vi and MenA saccharides, it was found that, depending on the antigen, saccharide length may or may not play a role [113]. Indeed, sugar length did not influence the MenA-specific serum IgG response (MenA OS of different and non-overlapping lengths—polymerization degrees equal to 5–12, 16–26, and >36—were tested), whereas longer Vi PS (48.5 kDa) were able to induce significantly higher Vi-specific serum IgGs than the shorter Vi OS (3.8 kDa). Interestingly, *E. coli* OMVs have been also genetically manipulated to express heterologous PSs of different lengths, thus resulting in glycoengineered OMVs (glyOMVs) [114,115,116].

## 5. Oligosaccharides from Monomers through Synthetic Approaches

Although isolation of PSs from bacterial growth is the primary approach for manufacturing licensed vaccines, short OS can also be chemically synthesized, and many reviews have recently summarized the progress of glycoconjugate vaccines based on synthetic carbohydrates [14,103,117]. Enzyme-catalyzed saccharide assembly has also been proposed as an interesting alternative, or as a supplement to, chemical synthesis for the generation of OS of interest, allowing stereo- and regio-selectivity control while avoiding complex protecting group manipulations [103]. Indeed, the synthesis of complex carbohydrates is still a challenging process as it requires multiple steps, the time-consuming purification of intermediates, and it provides relatively low overall yields. On the other hand, the use of structurally-defined synthetic OS avoids large-scale pathogen fermentation, and it improves batch-to-batch reproducibility and product characterization due to the conjugation process. As discussed above, the polydispersity of saccharide preparations, and the degree of overlap of their molecular size distributions, can complicate the investigation concerning the impact of sugar length on the immune response elicited by glycoconjugate vaccines. The availability of synthetic OS with defined chain lengths has facilitated elegant studies on the influence of saccharide chain length on conjugate immunogenicity [35,56]. For example, the use of structurally-defined synthetic *S. dysenteriae* type 1 OS allowed verification of the fact that chain length and saccharide loading on the carrier protein are interconnected parameters, and shorter chains need a higher density than longer ones to elicit an optimal antibody response [46]. The development of a synthetic *Shighella flexneri* 2a conjugate vaccine offers another example of the use of carbohydrate synthesis to select the optimal chain length; a conjugate made with a synthetic O-SP pentadecasaccharide, corresponding to 3 RU, was found to be more immunogenic than conjugates with 2 or 1 RU with the same saccharide load density [48]. This synthetic conjugate is currently in phase 2 clinical trials [118,119] and it constitutes clear evidence for the feasibility of this approach, together with the already commercialized synthetic Hib vaccine (Quimi-Hib, manufactured by CIGB, Cuba), which consists of an octamer end-linked to TT [120].

The use of synthetic fragments is increasingly used to identify minimal epitopes for antigen recognition [82,121], and the minimum chain length required to induce a strong immunological response is an important parameter for the design of synthetic glycoconjugate vaccines.

## 6. Discussion

Glycoconjugation is a well-established technology for the development of safe and effective vaccines, especially for children under the age of two. Traditionally, glycoconjugation has made use of shorter OS to overcome manufacturability issues that are encountered when using longer, native PSs, such as the high viscosity of saccharide solutions, difficulty in purifying the conjugate from free PSs, lack of a robust process, and manufacturing consistency issues; however, saccharide length is one of the main parameters affecting the immunogenicity of glycoconjugate vaccines, and unlike unconjugated PSs, antibody responses can be induced by very short OS after conjugation to a carrier protein [55,122,123,124,125]. 

For each antigen of interest, studies are therefore necessary to identify the optimal sugar length, as it is difficult to generalize the optimal length a priori, and other interconnected parameters, such as conjugation chemistry, carrier protein, saccharide loading, influence the immunogenicity of glycoconjugate vaccines. Overall, the use of long or short saccharide antigens seems to guide the selection of random versus selective conjugation approaches. A review of the literature suggests that, when using selective conjugation chemistries, the use of short- or medium-sized OS results in higher immunogenicity constructs than longer/native size PSs. In contrast, random chemistries can result in more immunogenic conjugate vaccines when longer PS chains are used. It can be hypothesized that, for random conjugates, long-chain PSs result in optimal saccharide epitope accessibility (which is not possible with shorter OS,), while avoiding T-independent activation of B cells due to appropriate crosslinking with the carrier protein. In contrast, selective end-linked conjugates containing long PSs may be able to directly activate B cells, whereas the use of shorter-sized OS can lead to appropriate antigen presentation and T cell dependent responses, provided that the minimum structural requirement for epitope recognition by the immune system is met [106].

Saccharide loading on the carrier protein appears to be particularly critical when using shorter OS, possibly due to the need to achieve an optimal antigen density for B-cell receptor recognition. Other variables are also important, including the presence of linkers or the antigenic properties.

Most of the published data on the influence of saccharide length on glycoconjugate-induced immunogenicity comes from animal studies. Few studies have been conducted in humans, so the question remains as to whether animal data can be predictive of clinical outcomes; however, similar results were obtained by comparing pneumococcal CRM_197_ conjugates in mice and human infants [28]. Studies in T-cell deficient and neonatal mice allowed the improvement of the immunogenicity of a Vi-CRM_197_ conjugate in humans [41]. In contrast, Hib OS of different lengths gave comparable results in rabbits and adults, but not in infants [53]. Making accurate predictions about vaccine efficacy in humans, and especially infants based on animal studies, remains difficult, and testing in more than one animal model could be valuable.

In this work, we have summarized the main methods used for PS fragmentation and sizing, highlighting the advantages and disadvantages of them all. Optimal methodologies should be selected based on the chemical structure of the target PS antigen and the desired OS size. Mild acidic hydrolysis has been widely used for its simplicity, scalability, and reproducibility. Similar principles apply to other hydrolysis methods (using hydrogen peroxide, ozone, nitrosonium cation, or periodate), but these require the presence of specific structures. Enzymatic hydrolysis is particularly recommended when the PS contains labile moieties (e.g., sialic acid side chains) as it only targets the enzyme-specific glycosidic linkage; however, enzymes may preferentially hydrolyze smaller saccharides, which may lead to low yields of saccharide fragments that are sufficiently long enough to elicit an appropriate immune response. Homogenization is one of the most widely used methods that produces OS for glycoconjugate vaccines. This method is simple, scalable, and without side reactions, but it is usually not suitable for production of very short OS. Sonication is another simple method to obtain OS, but is difficult to scale up, and may suffer from a lack of reproducibility. 

The use of synthetic fragments plays a critical role in identifying minimal epitopes for antigen recognition and the minimum saccharide size required to induce an effective immune response. Structurally-defined synthetic OS allows improved selectivity control to eliminate pathogen fermentation and improve both process reproducibility and product characterization; however, there are still economic, technical, and manufacturing limitations that hinder the use of synthetic approaches to produce complex and/or longer saccharide antigens. Studies are underway to understand the minimum epitope length that can be recognized by functional human monoclonal antibodies, and results may drive the design of well-defined OS obtained through chemical or/and enzymatic synthesis. 

Recent advancements in glycoengineering are also facilitating the generation of glycoconjugate vaccines by allowing the ad hoc modulation of the PS size. Bioconjugation allows the direct generation of the final conjugate, and it avoids the multiple steps of PS purification, fragmentation, and sizing before conjugation to the carrier protein. These innovative tools may play a role in facilitating an understanding of the impact of key parameters, such as sugar length, on the immunogenicity of glycoconjugate vaccines, and it may support the development of highly effective next-generation constructs.

## Terminology and Abbreviations

BSABovine serum albuminCPSCapsular polysaccharide(s)CRM_197_Cross-Reactive Material 197DxDextran(s)DTDiphtheria ToxoidGBSGroup B *Streptococcus*Hib*Hemophilus influenzae* type bMenMeningococcusMWMolecular weightO-SPO-specific polysaccharideOSOligosaccharide(s). This term will be used in this paper to indicate a saccharide fragment smaller than the native polysaccharidePRPPolyribosylribitol PhosphatePSPolysaccharide(s)RURepeating unit(s)TTTetanus toxoid

## Figures and Tables

**Table 1:** Methods to reduce PS size: target of the sugar structure, properties, and examples.

Method	Target	Properties	Examples *	References
Hydrolysis	Glycosidic O atom	Simple, scalable, and reproducible. Particularly useful when not all glycosidic linkages are cleaved at the same rate for homopolymers. It may impact the structural integrity of the RU.	Men CPS (A, C, W-135, Y, X), Hib CPS, *S. pneumoniae* CPS (3, 6A) (temperature-aided acid hydrolysis), Hib CPS (thermal hydrolysis)	[12,16,20,33,37,45,54,57,58,59,60,61,62,63,64,65,66]
Reactive oxygen and nitrogen species	C–H of Carbons involved in glycosidic bond (hydrogen peroxide) Anomeric Carbon (ozone) Amino or Sulfo-amino groups (nitrosonium cation)	Simple, scalable, and reproducible. Ozone targets mainly PS β-d-linkages whereas nitrosation requires a free amino or sulfo-amino group. Depending on the specific structure, this may or may not result in fragmentation.	Men CPS (A, C, W-135, Y), *S*. Typhi Vi CPS (hydrogen peroxide), GBS CPS (ozone), GBS CPS (II and III), *S. pneumoniae* CPS (14) (nitrosonium cation)	[26,31,41,42,67,68,69,70,71,72,73,74,75,76,77,78,79,80,81,82,83]
Periodate oxidation	Vicinal diols	Simple, scalable, and reproducible. Selectivity can be controlled by adjusting the oxidant concentration. It requires vicinal diols in specific positions (e.g., MenC CPS or Hib CPS) to lead to fragmentation, so this method cannot be generally applicable.	MenC CPS, Hib CPS, *S. pneumoniae* CPS (4)	[27,53,84,85,86,87,88,89,90,91]
Enzymatic hydrolysis	O-glycosidic linkages	Recommended for PS containing labile moieties (e.g., sialic acid side chains) as this only targets the enzyme-specific glycosidic linkage. Enzymes only hydrolyze specific substrates and may preferentially hydrolyze smaller saccharides.	GBS III CPS, *Klebsiella pneumoniae* CPS (K1 and K2), *S.* Typhimurium O-SP, Hib CPS	[23,24,26,43,54,92,93,94]
Homogenization	Mechanical sizing	Simple and scalable. Very reproducible and with no side reactions. Pressures needed to create very small OS may not be achievable.	*S. pneumoniae* CPS, Men CPS, GBS CPS, *Staphylococcus aureus* CPS	[20]
Sonication	Physical shearing of the PS via ultrasonic waves	Simple, but not easily scalable or reproducible. Generally, it does not induce side reactions, but the sonication probe may degrade and introduce metal contaminants into the PS solution.	*S. pneumoniae* CPS, *S*. Typhi Vi CPS, Hib CPS, *Staphylococcus aureus* CPS (5 and 8), *Cryptococcus neoformans* GXM CPS(A)	[20,38,95,96,97,98,99]

* The table refers to the discussed examples in the review.

## Data Availability

Not applicable.

## References

[1] Ovodov Y.S. (2006). Capsular antigens of bacteria. Capsular antigens as the basis of vaccines against pathogenic bacteria. Biochem. Biokhimiia.

[2] Micoli F., Costantino P., Adamo R. (2018). Potential targets for next generation anti-microbial glycoconjugate vaccines. FEMS Microbiol. Rev..

[3] Finland M., Sutliff W.D. (1932). Specific antibody response of human subjects to intracutaneous injection of pneumococcus products. J. Exp. Med..

[4] Macleod C.M., Hodges R.G., Heidelberger M., Bernhard W.G. (1945). Prevention of pneumococcal pneumonia by immunization with specific capsular polysaccharides. J. Exp. Med..

[5] Weintraub A. (2003). Immunology of bacterial polysaccharide antigens. Carbohydr. Res..

[6] Stefanetti G., Borriello F., Richichi B., Zanoni I., Lay L. (2021). Immunobiology of carbohydrates: Implications for novel vaccine and adjuvant design against infectious diseases. Front. Cell. Infect. Microbiol..

[7] Berti F., Micoli F. (2020). Improving efficacy of glycoconjugate vaccines: From chemical conjugates to next generation constructs. Curr. Opin. Immunol..

[8] Rappuoli R., De Gregorio E., Costantino P. (2019). On the mechanisms of conjugate vaccines. Proc. Natl. Acad. Sci. USA.

[9] Kelly D.F., Pollard A.J., Moxon E.R. (2005). Immunological memory: The role of B cells in long-term protection against invasive bacterial pathogens. JAMA.

[10] Avery O.T., Goebel W.F. (1929). Chemo-immunological studies on conjugated carbohydrate-proteins: II. Immunological specificity of synthetic sugar-protein antigens. J. Exp. Med..

[11] Goebel W.F., Avery O.T. (1931). Chemo-immunological studies on conjugated carbohydrate-proteins: IV. The synthesis of thep-aminobenzyl ether of the soluble specific substance of type III pneumococcus and its coupling with protein. J. Exp. Med..

[12] Schneerson R., Barrera O., Sutton A., Robbins J.B. (1980). Preparation, characterization, and immunogenicity of *Haemophilus influenzae* type b polysaccharide-protein conjugates. J. Exp. Med..

[13] Adamo R. (2021). Glycoconjugate vaccines: Classic and novel approaches. Glycoconj. J..

[14] Del Bino L., Østerlid K.E., Wu D.Y., Nonne F., Romano M.R., Codée J., Adamo R. (2022). Synthetic glycans to improve current glycoconjugate vaccines and fight antimicrobial resistance. Chem. Rev..

[15] Ravenscroft N., Jones C. (2000). Glycoconjugate vaccines. Curr. Opin. Drug Discov. Dev..

[16] Costantino P., Rappuoli R., Berti F. (2011). The design of semi-synthetic and synthetic glycoconjugate vaccines. Expert Opin. Drug Discov..

[17] Khatun F., Stephenson R.J., Toth I. (2017). An overview of structural features of antibacterial glycoconjugate vaccines that influence their immunogenicity. Chemistry.

[18] Micoli F., Adamo R., Costantino P. (2018). Protein carriers for glycoconjugate vaccines: History, selection criteria, characterization and new trends. Molecules.

[19] Stefanetti G., Hu Q.Y., Usera A., Robinson Z., Allan M., Singh A., Imase H., Cobb J., Zhai H., Quinn D. (2015). Sugar-protein connectivity impacts on the immunogenicity of site-selective *Salmonella* O-antigen glycoconjugate vaccines. Angew. Chem..

[20] Hennessey J.P., Costantino P., Talaga P., Beurret M., Ravenscroft N., Alderson M.R., Zablackis E., Prasad A.K., Frasch C. (2018). Lessons learned and future challenges in the design and manufacture of glycoconjugate vaccines. Am. Chem. Soc..

[21] Seppälä I., Mäkelä O. (1989). Antigenicity of dextran-protein conjugates in mice. Effect of molecular weight of the carbohydrate and comparison of two modes of coupling. J. Immunol..

[22] Fernandez C., Sverremark E. (1994). Immune responses to bacterial polysaccharides: Terminal epitopes are more immunogenic than internal structures. Cell. Immunol..

[23] Paoletti L.C., Kasper D.L., Michon F., DiFabio J., Holme K., Jennings H.J., Wessels M.R. (1990). An oligosaccharide-tetanus toxoid conjugate vaccine against type III group B Streptococcus. J. Biol. Chem..

[24] Paoletti L.C., Kasper D.L., Michon F., DiFabio J., Jennings H.J., Tosteson T.D., Wessels M.R. (1992). Effects of chain length on the immunogenicity in rabbits of group B Streptococcus type III oligosaccharide-tetanus toxoid conjugates. J. Clin. Investig..

[25] Wessels M.R., Paoletti L.C., Guttormsen H.K., Michon F., D’Ambra A.J., Kasper D.L. (1998). Structural properties of group B streptococcal type III polysaccharide conjugate vaccines that influence immunogenicity and efficacy. Infect. Immun..

[26] Michon F., Uitz C., Sarkar A., D’Ambra A.J., Laude-Sharp M., Moore S., Fusco P.C. (2006). Group B streptococcal type II and III conjugate vaccines: Physicochemical properties that influence immunogenicity. Clin. Vaccine Immunol..

[27] Peeters C.C., Tenbergen-Meekes A.M., Evenberg D.E., Poolman J.T., Zegers B.J., Rijkers G.T. (1991). A comparative study of the immunogenicity of pneumococcal type 4 polysaccharide and oligosaccharide tetanus toxoid conjugates in adult mice. J. Immunol..

[28] Daum R.S., Hogerman D., Rennels M.B., Bewley K., Malinoski F., Rothstein E., Reisinger K., Block S., Keyserling H., Steinhoff M. (1997). Infant immunization with pneumococcal CRM197 vaccines: Effect of saccharide size on immunogenicity and interactions with simultaneously administered vaccines. J. Infect. Dis..

[29] Laferriere C.A., Sood R.K., de Muys J.M., Michon F., Jennings H.J. (1997). The synthesis of *Streptococcus pneumoniae* polysaccharide-tetanus toxoid conjugates and the effect of chain length on immunogenicity. Vaccine.

[30] Arndt B., Porro M. (1991). Strategies for type-specific glycoconjugate vaccines of *Streptococcus pneumoniae*. Adv. Exp. Med. Biol..

[31] Laferriere C.A., Sood R.K., de Muys J.M., Michon F., Jennings H.J. (1998). *Streptococcus pneumoniae* type 14 polysaccharide-conjugate vaccines: Length stabilization of opsonophagocytic conformational polysaccharide epitopes. Infect. Immun..

[32] Pawlowski A., Källenius G., Svenson S.B. (2000). Preparation of pneumococcal capsular polysaccharide-protein conjugate vaccines utilizing new fragmentation and conjugation technologies. Vaccine.

[33] Pichichero M.E., Porcelli S., Treanor J., Anderson P. (1998). Serum antibody responses of weanling mice and two-year-old children to pneumococcal-type 6A-protein conjugate vaccines of differing saccharide chain lengths. Vaccine.

[34] Steinhoff M.C., Edwards K., Keyserling H., Thoms M.L., Johnson C., Madore D., Hogerman D. (1994). A randomized comparison of three bivalent *Streptococcus pneumoniae* glycoprotein conjugate vaccines in young children: Effect of polysaccharide size and linkage characteristics. Pediatr. Infect. Dis. J..

[35] Xiong C., Feng S., Qiao Y., Guo Z., Gu G. (2018). Synthesis and immunological studies of oligosaccharides that consist of the repeating unit of *Streptococcus pneumoniae* serotype 3 capsular polysaccharide. Chemistry.

[36] Carmenate T., Canaán L., Alvarez A., Delgado M., González S., Menéndez T., Rodés L., Guillén G. (2004). Effect of conjugation methodology on the immunogenicity and protective efficacy of meningococcal group C polysaccharide-P64k protein conjugates. FEMS Immunol. Med. Microbiol..

[37] Pietri G.P., Tontini M., Brogioni B., Oldrini D., Robakiewicz S., Henriques P., Calloni I., Abramova V., Santini L., Malić S. (2021). Elucidating the structural and minimal protective epitope of the serogroup X meningococcal capsular polysaccharide. Front. Mol. Biosci..

[38] Szu S.C., Li X.R., Schneerson R., Vickers J.H., Bryla D., Robbins J.B. (1989). Comparative immunogenicities of Vi polysaccharide-protein conjugates composed of cholera toxin or its B subunit as a carrier bound to high- or lower-molecular-weight Vi. Infect. Immun..

[39] Bhutta Z.A., Capeding M.R., Bavdekar A., Marchetti E., Ariff S., Soofi S.B., Anemona A., Habib M.A., Alberto E., Juvekar S. (2014). Immunogenicity and safety of the Vi-CRM197 conjugate vaccine against typhoid fever in adults, children, and infants in south and southeast Asia: Results from two randomised, observer-blind, age de-escalation, phase 2 trials. Lancet Infect. Dis..

[40] Van Damme P., Kafeja F., Anemona A., Basile V., Hilbert A.K., De Coster I., Rondini S., Micoli F., Qasim Khan R.M., Marchetti E. (2011). Safety, immunogenicity and dose ranging of a new Vi-CRM_197_ conjugate vaccine against typhoid fever: Randomized clinical testing in healthy adults. PLoS ONE.

[41] Micoli F., Bjarnarson S.P., Arcuri M., Aradottir Pind A.A., Magnusdottir G.J., Necchi F., Di Benedetto R., Carducci M., Schiavo F., Giannelli C. (2020). Short Vi-polysaccharide abrogates T-independent immune response and hyporesponsiveness elicited by long Vi-CRM_197_ conjugate vaccine. Proc. Natl. Acad. Sci. USA.

[42] Arcuri M., Di Benedetto R., Cunningham A.F., Saul A., MacLennan C.A., Micoli F. (2017). The influence of conjugation variables on the design and immunogenicity of a glycoconjugate vaccine against *Salmonella* Typhi. PLoS ONE.

[43] Svenson S.B., Lindberg A.A. (1981). Artificial *Salmonella* vaccines: *Salmonella typhimurium* O-antigen-specific oligosaccharide-protein conjugates elicit protective antibodies in rabbits and mice. Infect. Immun..

[44] Rondini S., Micoli F., Lanzilao L., Gavini M., Alfini R., Brandt C., Clare S., Mastroeni P., Saul A., MacLennan C.A. (2015). Design of glycoconjugate vaccines against invasive African *Salmonella enterica* serovar Typhimurium. Infect. Immun..

[45] Stefanetti G., Okan N., Fink A., Gardner E., Kasper D.L. (2019). Glycoconjugate vaccine using a genetically modified O antigen induces protective antibodies to Francisella tularensis. Proc. Natl. Acad. Sci. USA.

[46] Pozsgay V., Chu C., Pannell L., Wolfe J., Robbins J.B., Schneerson R. (1999). Protein conjugates of synthetic saccharides elicit higher levels of serum IgG lipopolysaccharide antibodies in mice than do those of the O-specific polysaccharide from *Shigella dysenteriae* type 1. Proc. Natl. Acad. Sci. USA.

[47] Robbins J.B., Kubler-Kielb J., Vinogradov E., Mocca C., Pozsgay V., Shiloach J., Schneerson R. (2009). Synthesis, characterization, and immunogenicity in mice of *Shigella sonnei* O-specific oligosaccharide-core-protein conjugates. Proc. Natl. Acad. Sci. USA.

[48] Phalipon A., Tanguy M., Grandjean C., Guerreiro C., Bélot F., Cohen D., Sansonetti P.J., Mulard L.A. (2009). A synthetic carbohydrate-protein conjugate vaccine candidate against *Shigella flexneri* 2a infection. J. Immunol..

[49] Kubler-Kielb J., Vinogradov E., Mocca C., Pozsgay V., Coxon B., Robbins J.B., Schneerson R. (2010). Immunochemical studies of *Shigella flexneri* 2a and 6, and *Shigella dysenteriae* type 1 O-specific polysaccharide-core fragments and their protein conjugates as vaccine candidates. Carbohydr. Res..

[50] Raso M.M., Gasperini G., Alfini R., Schiavo F., Aruta M.G., Carducci M., Forgione M.C., Martini S., Cescutti P., Necchi F. (2020). GMMA and glycoconjugate approaches compared in mice for the development of a vaccine against *Shigella flexneri* Serotype 6. Vaccines.

[51] Ftacek P., Nelson V., Szu S.C. (2013). Immunochemical characterization of synthetic hexa-, octa- and decasaccharide conjugate vaccines for *Vibrio cholerae* O:1 serotype Ogawa with emphasis on antigenic density and chain length. Glycoconj. J..

[52] Rana R., Dalal J., Singh D., Kumar N., Hanif S., Joshi N., Chhikara M.K. (2015). Development and characterization of *Haemophilus influenzae* type b conjugate vaccine prepared using different polysaccharide chain lengths. Vaccine.

[53] Anderson P.W., Pichichero M.E., Insel R.A., Betts R., Eby R., Smith D.H. (1986). Vaccines consisting of periodate-cleaved oligosaccharides from the capsule of *Haemophilus influenzae* type b coupled to protein carrier: Structural and temporal a requirements for priming in the human infant. J. Immunol..

[54] Anderson P.W., Pichichero M.E., Stein E.C., Porcelli S., Betts R.F., Connuck D.M., Korones D., Insel R.A., Zahradnik J.M., Eby R. (1989). Effect of oligosaccharide chain length, exposed terminal group, and hapten loading on the antibody response of human adults and infants to vaccines consisting of *Haemophilus influenzae* type b capsular antigen unterminally coupled to the diphtheria protein CRM197. J. Immunol..

[55] Peeters C.C., Evenberg D., Hoogerhout P., Käyhty H., Saarinen L., van Boeckel C.A., van der Marel G.A., van Boom J.H., Poolman J.T. (1992). Synthetic trimer and tetramer of 3-beta-D-ribose-(1-1)-D-ribitol-5-phosphate conjugated to protein induce antibody responses to *Haemophilus influenzae* type b capsular polysaccharide in mice and monkeys. Infect. Immun..

[56] Chong P., Chan N., Kandil A., Tripet B., James O., Yang Y.P., Shi S.P., Klein M. (1997). A strategy for rational design of fully synthetic glycopeptide conjugate vaccines. Infect. Immun..

[57] Lindberg B., Lönngren J., Svensson S., Tipson R.S., Horton D. (1975). Specific degradation of polysaccharides. Advances in Carbohydrate Chemistry and Biochemistry.

[58] Kamerling J.P., Gerwig G.J. (2007). Strategies for the structural analysis of carbohydrates. Compr. Glycosci..

[59] Broker M., Dull P.M., Rappuoli R., Costantino P. (2009). Chemistry of a new investigational quadrivalent meningococcal conjugate vaccine that is immunogenic at all ages. Vaccine.

[60] Costantino P., Norelli F., Giannozzi A., D’Ascenzi S., Bartoloni A., Kaur S., Tang D., Seid R., Viti S., Paffetti R. (1999). Size fractionation of bacterial capsular polysaccharides for their use in conjugate vaccines. Vaccine.

[61] Beurret M., Hamidi A., Kreeftenberg H. (2012). Development and technology transfer of *Haemophilus influenzae* type b conjugate vaccines for developing countries. Vaccine.

[62] Hitri K., Kuttel M.M., De Benedetto G., Lockyer K., Gao F., Hansal P., Rudd T.R., Beamish E., Rijpkema S., Ravenscroft N. (2019). O-acetylation of typhoid capsular polysaccharide confers polysaccharide rigidity and immunodominance by masking additional epitopes. Vaccine.

[63] Costantino P., Viti S., Podda A., Velmonte M.A., Nencioni L., Rappuoli R. (1992). Development and phase 1 clinical testing of a conjugate vaccine against meningococcus A and C. Vaccine.

[64] Berti F., Romano M.R., Micoli F., Adamo R. (2021). Carbohydrate based meningococcal vaccines: Past and present overview. Glycoconj. J..

[65] Matjila M.J., Phohu T.C., Banzhoff A., Viviani S., Hoosen A.A., Bianchini M., Nacci P., Palweni C.W., Podda A., Whitfield M. (2004). Safety and immunogenicity of two *Haemophilus influenzae* type b conjugate vaccines. S. Afr. Med. J..

[66] Lefeber D.J., Gutiérrez Gallego R., Grün C.H., Proietti D., D’Ascenzi S., Costantino P., Kamerling J.P., Vliegenthart J.F. (2002). Isolation of oligosaccharides from a partial-acid hydrolysate of pneumococcal type 3 polysaccharide for use in conjugate vaccines. Carbohydr. Res..

[67] Duan J., Kasper D.L. (2011). Oxidative depolymerization of polysaccharides by reactive oxygen/nitrogen species. Glycobiology.

[68] Kohen R., Nyska A. (2002). Oxidation of biological systems: Oxidative stress phenomena, antioxidants, redox reactions, and methods for their quantification. Toxicol. Pathol..

[69] Ofoedu C.E., You L., Osuji C.M., Iwouno J.O., Kabuo N.O., Ojukwu M., Agunwah I.M., Chacha J.S., Muobike O.P., Agunbiade A.O. (2021). Hydrogen peroxide effects on natural-sourced polysacchrides: Free radical formation/production, degradation process, and reaction mechanism-A critical synopsis. Foods.

[70] Hawkins C.L., Davies M.J. (1996). Direct detection and identification of radicals generated during the hydroxyl radical-induced degradation of hyaluronic acid and related materials. Free Radic. Biol. Med..

[71] Gilbert B.C., King D.M., Thomas C.B. (1981). Radical reactions of carbohydrates. Part 2. An electron spin resonance study of the oxidation of D-glucose and related compounds with the hydroxyl radical. J. Chem. Soc. Perkin Trans..

[72] Rees M.D., Kennett E.C., Whitelock J.M., Davies M.J. (2008). Oxidative damage to extracellular matrix and its role in human pathologies. Free Radic. Biol. Med..

[73] Wang N., Zhang X., Wang S., Guo Q., Li Z., Liu H., Wang C. (2020). Structural characterisation and immunomodulatory activity of polysaccharides from white asparagus skin. Carbohydr. Polym..

[74] Ryall R.P. (2004). Multivalent Meningococcal Derivatized Polysaccharide-Protein Conjugates and Vaccines. U.S. Patent.

[75] Vreeburg R.A., Airianah O.B., Fry S.C. (2014). Fingerprinting of hydroxyl radical-attacked polysaccharides by N-isopropyl-2-aminoacridone labelling. Biochem. J..

[76] Cai X., Lei Q.P., Lamb D.H., Shannon A., Jacoby J., Kruk J., Kensinger R.D., Ryall R., Zablackis E., Cash P. (2004). LC/MS characterization of meningococcal depolymerized polysaccharide group C reducing endgroup and internal repeating unit. Anal. Chem..

[77] Wang Y., Hollingsworth R.I., Kasper D.L. (1998). Ozonolysis for selectively depolymerizing polysaccharides containing beta-D-aldosidic linkages. Proc. Natl. Acad. Sci. USA.

[78] Wang Y., Hollingsworth R.I., Kasper D.L. (2001). Method for Generating Antibodies to Saccharide Fragments. U.S. Patent.

[79] Wang J.Y., Chang A.H., Guttormsen H.K., Rosas A.L., Kasper D.L. (2003). Construction of designer glycoconjugate vaccines with size-specific oligosaccharide antigens and site-controlled coupling. Vaccine.

[80] Lindberg B., Lindqvist B., Lönngren J., Powell D.A. (1980). Structural studies of the capsular polysaccharide from *Streptococcus pneumoniae* type 1. Carbohydr. Res..

[81] Kenne L., Lindberg B., Unger P., Gustafsson B., Holme T. (1982). Structural studies of the *Vibrio cholerae* O-antigen. Carbohydr. Res..

[82] Carboni F., Adamo R., Fabbrini M., De Ricco R., Cattaneo V., Brogioni B., Veggi D., Pinto V., Passalacqua I., Oldrini D. (2017). Structure of a protective epitope of group B Streptococcus type III capsular polysaccharide. Proc. Natl. Acad. Sci. USA.

[83] Zou W., Laferriere C.A., Jennings H.J. (1998). Oligosaccharide fragments of the type III group B streptococcal polysaccharide derived from *S. pneumoniae* type 14 capsular polysaccharide by a chemoenzymatic method. Carbohydr. Res..

[84] Van Lenten L., Ashwell G. (1971). Studies on the chemical and enzymatic modification of glycoproteins. A general method for the tritiation of sialic acid-containing glycoproteins. J. Biol. Chem..

[85] Wilchek M., Bayer E.A. (1987). Labeling glycoconjugates with hydrazide reagents. Methods Enzymol..

[86] Lotan R., Debray H., Cacan M., Cacan R., Sharons N. (1975). Labeling of soybean agglutinin by oxidation with sodium periodate followed by reduction with sodium [3-H] borohydride. J. Biol. Chem..

[87] Ouellette R.J., Rawn J.D., Ouellette R.J., Rawn J.D. (2018). 16-alcohols: Reactions and synthesis. Organic Chemistry.

[88] Stefanetti G., Rondini S., Lanzilao L., Saul A., MacLennan C.A., Micoli F. (2014). Impact of conjugation chemistry on the immunogenicity of *S. Typhimurium* conjugate vaccines. Vaccine.

[89] Zou W., Jennings H.J., Guo Z., Boons G.-J. (2009). Preparation of glycoconjugate vaccines. Carbohydrate-Based Vaccines and Immunotherapies 2009.

[90] Anderson P.W. (1983). Immunogenic Conjugates. U.S. Patent.

[91] Jennings H.J., Lugowski C. (1981). Immunochemistry of groups A, B, and C meningococcal polysaccharide-tetanus toxoid conjugates. J. Immunol..

[92] Rollings J. (1985). Enzymatic fragmentation of polysaccharides. Carbohydr. Polym..

[93] Paoletti L.C., Johnson K.D. (1995). Purification of preparative quantities of group B Streptococcus type III oligosaccharides. J. Chromatogr. A.

[94] Lin T.-L., Yang F.-L., Ren C.-T., Pan Y.-J., Liao K.-S., Tu I.-F., Chang Y.-P., Cheng Y.-Y., Wu C.-Y., Wu S.-H. (2022). Development of klebsiella pneumoniae capsule polysaccharide-conjugated vaccine candidates using phage depolymerases. Front. Immunol..

[95] Turner A.E.B., Gerson J.E., So H.Y., Krasznai D.J., St Hilaire A.J., Gerson D.F. (2017). Novel polysaccharide-protein conjugates provide an immunogenic 13-valent pneumococcal conjugate vaccine for *S. pneumoniae*. Synth. Syst. Biotechnol..

[96] Szu S.C., Zon G., Schneerson R., Robbins J.B. (1986). Ultrasonic irradiation of bacterial polysaccharides. Characterization of the depolymerized products and some applications of the process. Carbohydr. Res..

[97] Fattom A., Shiloach J., Bryla D., Fitzgerald D., Pastan I., Karakawa W.W., Robbins J.B., Schneerson R. (1992). Comparative immunogenicity of conjugates composed of the *Staphylococcus aureus* type 8 capsular polysaccharide bound to carrier proteins by adipic acid dihydrazide or N-succinimidyl-3-(2-pyridyldithio)propionate. Infect. Immun..

[98] Fattom A., Schneerson R., Watson D.C., Karakawa W.W., Fitzgerald D., Pastan I., Li X., Shiloach J., Bryla D.A., Robbins J.B. (1993). Laboratory and clinical evaluation of conjugate vaccines composed of *Staphylococcus aureus* type 5 and type 8 capsular polysaccharides bound to Pseudomonas aeruginosa recombinant exoprotein A. Infect. Immun..

[99] Devi S.J., Schneerson R., Egan W., Ulrich T.J., Bryla D., Robbins J.B., Bennett J.E. (1991). Cryptococcus neoformans serotype A glucuronoxylomannan-protein conjugate vaccines: Synthesis, characterization, and immunogenicity. Infect. Immun..

[100] Wacker M., Linton D., Hitchen P.G., Nita-Lazar M., Haslam S.M., North S.J., Panico M., Morris H.R., Dell A., Wren B.W. (2002). N-linked glycosylation in *Campylobacter jejuni* and its functional transfer into *E. coli*. Science.

[101] Feldman M.F., Wacker M., Hernandez M., Hitchen P.G., Marolda C.L., Kowarik M., Morris H.R., Dell A., Valvano M.A., Aebi M. (2005). Engineering N-linked protein glycosylation with diverse O antigen lipopolysaccharide structures in *Escherichia coli*. Proc. Natl. Acad. Sci. USA.

[102] Faridmoayer A., Fentabil M.A., Mills D.C., Klassen J.S., Feldman M.F. (2007). Functional characterization of bacterial oligosaccharyltransferases involved in O-linked protein glycosylation. J. Bacteriol..

[103] Micoli F., Del Bino L., Alfini R., Carboni F., Romano M.R., Adamo R. (2019). Glycoconjugate vaccines: Current approaches towards faster vaccine design. Expert Rev. Vaccines.

[104] Harding C.M., Feldman M.F. (2019). Glycoengineering bioconjugate vaccines, therapeutics, and diagnostics in *E. coli*. Glycobiology.

[105] Wacker M., Wang L., Kowarik M., Dowd M., Lipowsky G., Faridmoayer A., Shields K., Park S., Alaimo C., Kelley K.A. (2014). Prevention of *Staphylococcus aureus* infections by glycoprotein vaccines synthesized in *Escherichia coli*. J. Infect. Dis..

[106] Anish C., Beurret M., Poolman J. (2021). Combined effects of glycan chain length and linkage type on the immunogenicity of glycoconjugate vaccines. NPJ Vaccines.

[107] Hegerle N., Bose J., Ramachandran G., Galen J.E., Levine M.M., Simon R., Tennant S.M. (2018). Overexpression of O-polysaccharide chain length regulators in Gram-negative bacteria using the Wzx-/Wzy-dependent pathway enhances production of defined modal length O-polysaccharide polymers for use as haptens in glycoconjugate vaccines. J. Appl. Microbiol..

[108] Kalynych S., Ruan X., Valvano M.A., Cygler M. (2011). Structure-guided investigation of lipopolysaccharide O-antigen chain length regulators reveals regions critical for modal length control. J. Bacteriol..

[109] Gasperini G., Raso M.M., Arato V., Aruta M.G., Cescutti P., Necchi F., Micoli F. (2021). Effect of O-antigen chain length regulation on the immunogenicity of *Shigella* and *Salmonella* generalized modules for membrane antigens (GMMA). Int. J. Mol. Sci..

[110] Micoli F., Alfini R., Di Benedetto R., Necchi F., Schiavo F., Mancini F., Carducci M., Palmieri E., Balocchi C., Gasperini G. (2020). GMMA is a versatile platform to design effective multivalent combination vaccines. Vaccines.

[111] Micoli F., MacLennan C.A. (2020). Outer membrane vesicle vaccines. Semin. Immunol..

[112] Piccioli D., Bartolini E., Micoli F. (2022). GMMA as a ‘plug and play’ technology to tackle infectious disease to improve global health: Context and perspectives for the future. Expert Rev. Vaccines.

[113] Micoli F., Alfini R., Di Benedetto R., Necchi F., Schiavo F., Mancini F., Carducci M., Oldrini D., Pitirollo O., Gasperini G. (2021). Generalized modules for membrane antigens as carrier for polysaccharides: Impact of sugar length, density, and attachment site on the immune response elicited in animal models. Front. Immunol..

[114] Chen L., Valentine J.L., Huang C.-J., Endicott C.E., Moeller T.D., Rasmussen J.A., Fletcher J.R., Boll J.M., Rosenthal J.A., Dobruchowska J. (2016). Outer membrane vesicles displaying engineered glycotopes elicit protective antibodies. Proc. Natl. Acad. Sci. USA.

[115] Price N.L., Goyette-Desjardins G., Nothaft H., Valguarnera E., Szymanski C.M., Segura M., Feldman M.F. (2016). Glycoengineered outer membrane vesicles: A novel platform for bacterial vaccines. Sci. Rep..

[116] Stevenson T.C., Cywes-Bentley C., Moeller T.D., Weyant K.B., Putnam D., Chang Y.-F., Jonese B.D., Pierb G.B., DeLisa M.P. (2018). Immunization with outer membrane vesicles displaying conserved surface polysaccharide antigen elicits broadly antimicrobial antibodies. Proc. Natl. Acad. Sci. USA.

[117] Colombo C., Pitirollo O., Lay L. (2018). Recent advances in the synthesis of glycoconjugates for vaccine development. Molecules.

[118] Van der Put R.M.F., Smitsman C., de Haan A., Hamzink M., Timmermans H., Uittenbogaard J., Westdijk J., Stork M., Ophorst O., Thouron F. (2022). The first-in-human synthetic glycan-based conjugate vaccine candidate against *Shigella*. ACS Cent. Sci..

[119] Phalipon A., Mulard L.A. (2022). Toward a multivalent synthetic oligosaccharide-based conjugate vaccine against *Shigella*: State-of-the-art for a monovalent prototype and challenges. Vaccines.

[120] Verez-Bencomo V., Fernandez-Santana V., Hardy E., Toledo M.E., Rodriguez M.C., Heynngnezz L., Rodriguez A., Baly A., Herrera L., Izquierdo M. (2004). A synthetic conjugate polysaccharide vaccine against *Haemophilus influenzae* type b. Science.

[121] Kenfack M.T., Mazur M., Nualnoi T., Shaffer T.L., Ngassimou A., Bleriot Y., Marrot J., Marchetti R., Sintiprungrat K., Chantratita N. (2017). Deciphering minimal antigenic epitopes associated with *Burkholderia pseudomallei* and *Burkholderia mallei* lipopolysaccharide O-antigens. Nat. Commun..

[122] Dalal J., Rana R., Harale K., Hanif S., Kumar N., Singh D., Chhikara M.K. (2019). Development and pre-clinical evaluation of a synthetic oligosaccharide-protein conjugate vaccine against *Neisseria meningitidis* serogroup C. Vaccine.

[123] Safari D., Dekker H.A., Joosten J.A., Michalik D., de Souza A.C., Adamo R., Lahmann M., Sundgren A., Oscarson S., Kamerling J.P. (2008). Identification of the smallest structure capable of evoking opsonophagocytic antibodies against *Streptococcus pneumoniae* type 14. Infect. Immun..

[124] Enotarpi J., Tontini M., Balocchi C., van der Es D., Auberger L., Balducci E., Carboni F., Proietti D., Casini D., Filippov D.V. (2020). A stabilized glycomimetic conjugate vaccine inducing protective antibodies against *Neisseria meningitidis* serogroup A. Nat. Commun..

[125] Phalipon A., Costachel C., Grandjean C., Thuizat A., Guerreiro C., Tanguy M., Nato F., Vulliez-Le Normand B., Bélot F., Wright K. (2006). Characterization of functional oligosaccharide mimics of the *Shigella flexneri* serotype 2a O-antigen: Implications for the development of a chemically defined glycoconjugate vaccine. J. Immunol..

